# Dysregulated acetylcholine-mediated dopamine neurotransmission in the eIF4E Tg mouse model of autism spectrum disorders

**DOI:** 10.1016/j.celrep.2024.114997

**Published:** 2024-11-27

**Authors:** Josep Carbonell-Roig, Alina Aaltonen, Karin Wilson, Maya Molinari, Veronica Cartocci, Avery McGuirt, Eugene Mosharov, Jan Kehr, Ori J. Lieberman, David Sulzer, Anders Borgkvist, Emanuela Santini

**Affiliations:** 1 Department of Neuroscience, Karolinska Institute, 17177 Stockholm, Sweden; 2 Department of Psychiatry, Columbia University Irving Medical Center, New York, NY 10032, USA; 3 New York State Psychiatric Institute, New York, NY 10032, USA; 4 Pronexus Analytical AB, 16733 Stockholm-Bromma, Sweden; 5 Department of Neurology, University of California San Francisco (UCSF), San Francisco, CA 94143, USA; 6 Lead contact

## Abstract

Autism spectrum disorder (ASD) consists of diverse neurodevelopmental conditions where core behavioral symptoms are critical for diagnosis. Altered dopamine (DA) neurotransmission in the striatum has been suggested to contribute to the behavioral features of ASD. Here, we examine DA neurotransmission in a mouse model of ASD characterized by elevated expression of eukaryotic initiation factor 4E (eIF4E), a key regulator of cap-dependent translation, using a comprehensive approach that encompasses genetics, behavior, synaptic physiology, and imaging. The results indicate that increased eIF4E expression leads to behavioral inflexibility and impaired striatal DA release. The loss of normal DA neurotransmission is due to a defect in nicotinic receptor signaling that regulates calcium dynamics in dopaminergic axons. These findings provide a mechanistic understanding of ASD symptoms and offer a foundation for targeted therapeutic interventions by revealing the intricate interplay between eIF4E, DA neurotransmission, and behavioral flexibility.

## In brief

Basal ganglia dysfunction potentially causes behavioral alterations in ASD. Carbonell-Roig et al. show that the eIF4E Tg mouse model of ASD exhibits behavioral inflexibility and impaired striatal dopamine release, linked to disrupted β2-nAChR function on DA axons. Thus, altered striatal ACh-DA interactions constitute a cellular basis for ASD-like behavioral inflexibility.

## Graphical Abstract

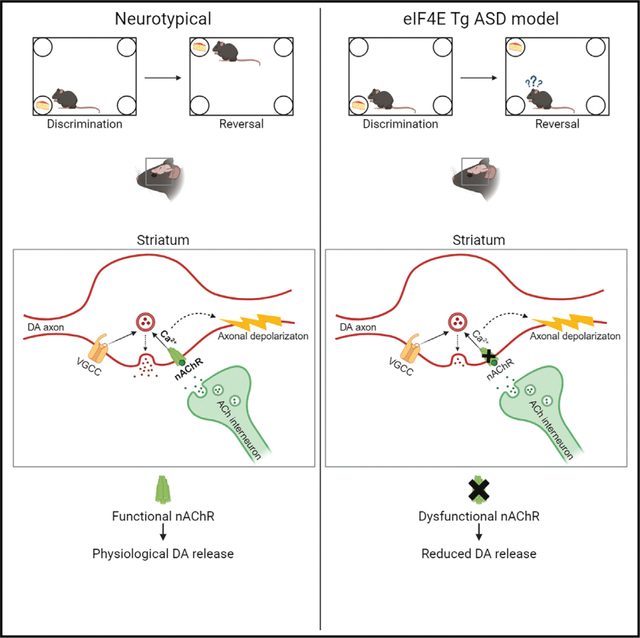

## INTRODUCTION

Autism spectrum disorder (ASD) encompasses a diverse set of polygenic neurodevelopmental conditions defined by core behavioral symptoms, such as repetitive, stereotyped behaviors and abnormal social interactions.^1^ The behavioral manifestations within ASD are heterogeneous and are often complicated by comorbidities. Thus, the clinical diagnosis of ASD is challenging, since it primarily relies on identification of this complex behavioral symptomatology.^2^

Emerging clinical and preclinical research aimed at determining brain alterations associated with ASD symptomatology suggests that the behavioral manifestations of ASD may stem from altered synaptic functions in the striatum, the main receiving nucleus of the basal ganglia.^3–5^ Dopamine (DA) neurotransmission is crucial for striatal physiology, as it plays a key role in selecting and transmitting motor patterns and contributes to reward-based learning, motivation, and salience. Disrupted DA signaling has been linked to hyperactivity, inattention, and stereotyped behaviors found in several neurodevelopmental disorders.^6–8^ Notably, abnormal DA signaling is observed in patients with ASD and animal models,^6–9^ although the precise mechanisms linking altered DA function and ASD symptomatology remain a subject of ongoing research.

A key feature of DA neurotransmission is that it is governed by both the spiking activity of midbrain DA neurons and local modulatory mechanisms in axons.^10–12^ In the striatum, several intrinsic and extrinsic mechanisms have been shown to control local DA release, with acetylcholine (ACh) being one of the most significant.^10–16^ Striatal ACh is released by tonically active interneurons and regulates DA release probability by binding to nicotinic ACh receptors (nAChRs) on the DA axons.^10,11,17–20^ DA axons express heteropentameric β2-subunit-containing nAChRs (β2-nAChRs),^21^ which are nonselective cation channels.^10,11,19–21^ Activation of β2-nAChRs initiates ectopic action potentials in DA axons, followed by the opening of voltage-gated Ca^2+^ channels (VGCCs) and DA release.^13,22,23^ Studies on axonal β2-nAChRs, including pharmacological manipulation and genetic deletion, have shown that these receptors act as high-pass filters^19,20,24,25^ enabling more DA release during high levels of synaptic activity. Inhibition or rapid desensitization of β2-nAChRs initially reduces DA release probability, diminishing DA release evoked by single stimuli but facilitating it with high-frequency activity.^10–12,19,20,24–27^ Therefore, dynamic changes in striatal ACh levels enhance local DA signals, especially when DA neurons switch from tonic to phasic firing in response to salient stimuli.

Syndromic^28–32^ and idiopathic^33–36^ forms of ASD have been linked to dysregulated mammalian target of rapamycin (mTOR) signaling, which regulates essential biochemical processes, including activity-dependent protein synthesis.^28,37–40^ Consistent with evidence showing that altered mTOR signaling and cap-dependent translation are pathogenic molecular alterations shared by ASD, mutations in the *EIF4E* gene, which encodes for the cap-binding protein eIF4E, and genetic variants on chromosome 4q, where this gene is located, have been linked to ASD.^32,41–43^

The central role of eIF4E-dependent translation in ASD has been demonstrated with preclinical models. Mice with global (referred to as eIF4E transgenic [Tg])^44^ or microglia-specific^45^ overexpression of Eif4e as well as constitutive genetic deletion of *4E-bp2*,^46^, a repressor of eIF4E, showed that aberrant brain eIF4E-dependent translation is associated with ASD-like behaviors and synaptic and structural abnormalities consistent with ASD pathology.

Here, we took advantage of the eIF4E Tg mice^44^ to test the hypothesis that behavioral inflexibility is linked to defective DA neurotransmission. By employing a combination of genetics, synaptic physiology, and imaging methods, we demonstrate that increased eIF4E expression results in reduced DA release in the striatum. Furthermore, our investigation identified a mechanism reliant on impaired nAChRs and axonal Ca^2+^ dynamics. Here, we provide evidence indicating that altered striatal DA neurotransmission plays a role as a pathological mechanism in ASD symptomatology.

## RESULTS

### The eIF4E Tg mice exhibit aberrant reversal learning

Striatal DA is implicated in cognitive flexibility,^47–49^ a feature commonly compromised in patients with ASD.^47,50–52^ Our previous studies have shown that the eIF4E Tg mice exhibit impaired reversal learning and cognitive inflexibility in behavioral tasks based on negative reinforcement; for instance, avoiding a punishment such as cold water or foot shock.^44^ However, it remains unknown whether the behavioral inflexibility of the eIF4E Tg mice extends to their ability to adapt to novel contingencies for the purpose of achieving positive outcomes, such as rewards.

**Figure 1.**
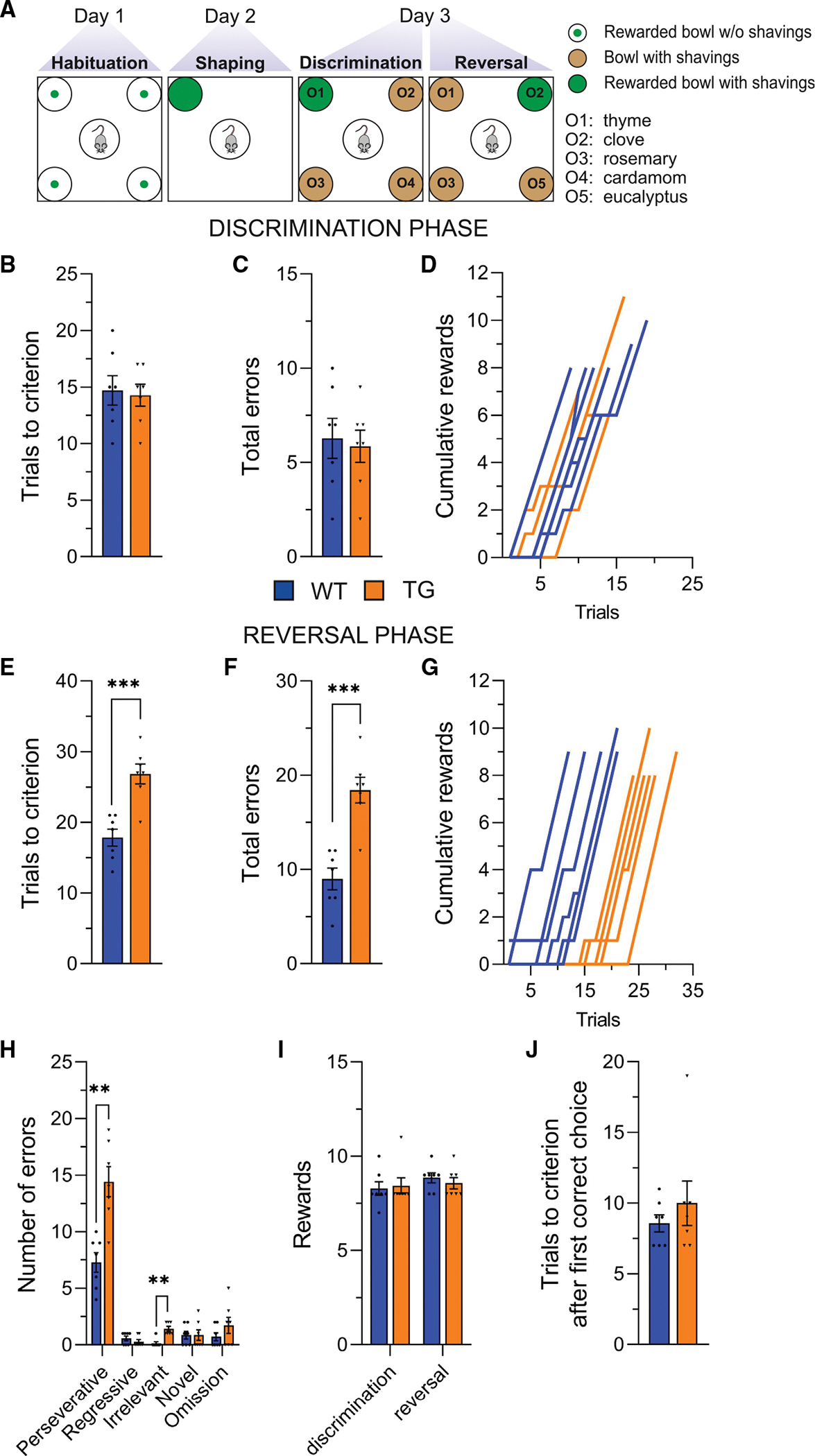
Cognitive inflexibility of the eIF4E Tg mice in the four-choice odor discrimination task (A) Task schematic. (B–D) Discrimination phase. Trials to criterion (B, eight of 10), (C) total errors, and (D) cumulative rewards during discrimination. Unpaired two-tailed t test: (B) t_12_ = 0.3, not significant (ns) and (C) t_12_ = 0.3, ns. (E–G) Reversal phase. Trials to criterion (E), total errors (F), and cumulative rewards during reversal (G). Unpaired two-tailed t test: (E) t_12_ = 4.9, ***p < 0.001 and (F) t_12_ = 5.3, ***p < 0.001. (H) Error types during reversal: perseverative, regressive, irrelevant, novel, and omission (STAR Methods). Two-way RM ANOVA, genotype: F_(1, 12)_ = 26.8, ***p < 0.001; error types: F_(4, 48)_ = 108.5, ****p < 0.0001; interaction genotype:error types: F_(4, 48)_ = 12.42, *p* < 0.0001; **p < 0.01, Bonferroni’s multiple-comparisons test. (I) Food rewards obtained during discrimination and reversal. Unpaired two-tailed t test, t_12_ = 0.3, ns (discrimination) and t_12_ = 0.7, ns (reversal). (J) Trials to criterion after the first correct choice. Unpaired two-tailed t test for discrimination, t_12_ = 0.8, ns. Bars represent mean ± SEM, and dots represent individual mice. Statistics for non-significant data are shown in Table S1. n = 7 mice/genotype.

To address this question, we employed the four-choice odor discrimination task,^53,54^ a reversal learning paradigm based on a naturalistic reward (e.g., food), which is sensitive to changes in DA neurotransmission.^55,56^ In this task, the mice initially learn to associate a food reward with a specific odor (O1-food in the discrimination phase; Figure 1A). The learned association is then challenged in the subsequent reversal phase, where the food is paired with a different odor (O2-food in the reversal phase; Figure 1A).

The eIF4E Tg mice displayed intact acquisition learning, as demonstrated by a similar number of trials required to reach criterion (eight successful food retrievals of 10; Figure 1B) and a similar number of errors (Figure 1C) compared to their wild-type (WT) littermates. Moreover, plots illustrating the cumulative rewards obtained by the mice over the different trials indicated that the curves for both WT and eIF4E Tg mice overlapped (Figure 1D). In contrast, during the reversal phase, when the food was associated with a different odor (O2-food), the eIF4E Tg mice required a significantly higher number of trials to reach criterion (Figure 1E), made more errors (Figure 1F), and displayed a distinct, right-shifted, non-overlapping cumulative reward curve (Figure 1G) compared to the WT mice. Notably, perseverative errors were the most frequent error type for all mice in the reversal phase of the test, but the eIF4E Tg mice exhibited significantly more perseverative errors than their WT littermates (Figure 1H). These results indicate that eIF4E Tg mice have impaired behavioral adaptation in response to altered environmental conditions.

To determine whether hunger status impacted the performance of the eIF4E Tg mice in the four-choice odor discrimination task, we examined the total number of food rewards obtained during the task and the trials needed to reach criterion after their first correct choice. The results showed no significant differences between the eIF4E Tg and WT control mice in both discrimination and reversal phases regarding the total food rewards (Figure 1I) and the trials to meet criterion after the first correct choice (Figure 1J). Overall, these findings suggest that hunger status is an unlikely factor contributing to the behavioral inflexibility of the eIF4E Tg mice.

In summary, the eIF4E Tg mice show inflexible behavior, as evidenced by increased number of trials needed to adapt a learned behavior in response to changes in the environment.

### Reduced DA release in the dorsal striatum of eIF4E Tg mice

The eIF4E Tg mice exhibited altered reversal learning in the four-choice odor discrimination task. Similar results have been shown previously to be dependent on altered DA release, excitation-inhibition balance, firing pattern, and morphology of DA neurons.^55,56^ Therefore, we hypothesized that eIF4E Tg mice would exhibit deficits in dopaminergic function. We sampled sub-second DA transients using Fast-Scan Cyclic Voltammetry (FSCV) at carbon fiber microelectrodes following local electrical stimulation of acute striatal slices from the eIF4E Tg mice and their WT littermates. We analyzed the dorsal striatum due to its involvement in reversal learning,^57–59^ particularly in the four-choice odor discrimination task.^55^ In addition to single-pulse stimulation, we also employed train stimuli (5 pulses at 10 Hz and 100 Hz [5p10Hz and 5p100Hz, respectively]) covering a range that emulates phasic firing of DA neurons and falls within the range that maximizes striatal DA release.^60^

**Figure 2.**
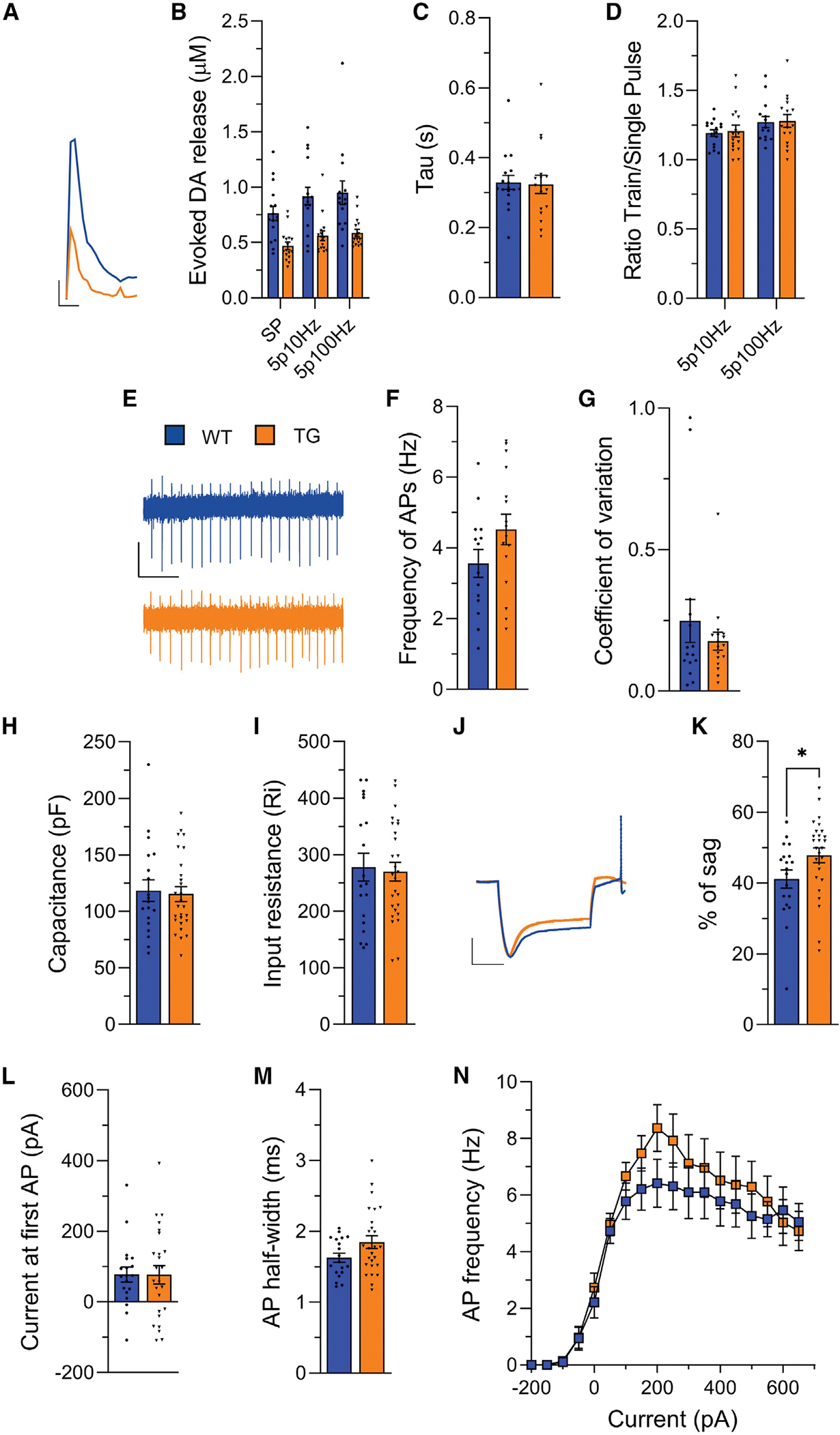
Altered striatal DA release but intact intrinsic properties and excitability of midbrain DA neurons of the eIF4E Tg mice (A) Representative FSCV traces with single-pulse stimulation (SP). Scale: 500 ms (x) and 0.1 μM (y). (B) Peak concentrations of DA release evoked by SP or 5 pulses at 10 Hz (5p10Hz) and 100 Hz (5p100Hz). Two-way RM ANOVA, genotype: F_(1, 30)_ = 15.99, ***p < 0.001; stimulation: F_(1.6, 48.3)_ = 32.18, ****p < 0.0001; interaction genotype:stimulation: F_(2, 58)_ = 2.58, *p* = 0.08. (C) Decay constant (tau) of SP-evoked DA transients. Unpaired two-tailed t test, t_28_ = 0.5, ns. (D) Ratio of peak DA release evoked by 5p10Hz and 5p100Hz over SP. Unpaired two-tailed t test, t_28_ = 0.04, ns. n = 14–17 slices/3–6 mice/genotype. (E) Representative cell-attached traces. Scale: 1 s (x) and 50 mV (y). (F) Firing frequency. Unpaired two-tailed t test, t_30_ = 1.377, ns. (G) Coefficient of variation of interspike intervals. Unpaired two-tailed t test, t_30_ = 0.9018, ns. (H) Capacitance. Unpaired two-tailed t test, t_44_ = 0.27, ns. (I) Input resistance. Unpaired two-tailed t test, t_44_ = 0.27, ns. (J) Representative voltage responses to hyperpolarizing current injection (300 pA). Scale: 200 ms (x) and −35 mV (y). (K) Ratio of the instantaneous versus steady-state voltage response to −300-pA current injection (% of sag). Unpaired two-tailed t test, t_44_ = 2.1, *p < 0.05. (L) Current at the first action potential (AP). Unpaired two-tailed t test, t_42_ = 0.016, ns. (M) AP half-width. Unpaired two-tailed t test, t_43_ = 1.86, ns. (N) Rate of AP (Hz) in response to a series of current steps of increasing amplitude. Two-way repeated measures (RM) ANOVA, genotype: F_(1, 44)_ = 0.56, *p* = 0.458, ns; current steps: F_(2.649, 116.6)_ = 66.2, ****p < 0.0001; interaction genotype:current steps: F_(19, 836)_ = 0.84, P = 0.66 ns. n = 15–27 neurons/3 mice/genotype. Bars represent mean ± SEM, and dots represent individual slices (A–D) or neurons (E–N). Statistics for non-significant data are shown in Table S2.

Both single-pulse and train stimulations revealed a significant reduction in the peak amplitude of DA efflux in the dorsal striatum of eIF4E Tg mice (Figures 2A and 2B).

The decrease in DA amplitude was not a result of increased reuptake, as the decay constant (tau) of the DA peak, which is influenced by the efficacy of the DA reuptake transporter (DAT),^61^ was similar in both WT and eIF4E Tg mice (Figure 2C).

We found that DA efflux is dynamically regulated by increasing the stimulation in both genotypes, as indicated by the similar train/single-pulse ratio (Figure 2D). Since high-frequency stimulation increases DA release by enhancing net axonal Ca^2+^ influx through VGCCs,^60,62^ our results indicate that there is no alteration in activity-dependent DA release related to major deficits in Ca^2+^ influx dynamics.

To examine whether the reduced DA release in eIF4E Tg depends on altered regulation by D2 receptors (D2Rs) we applied the D2R-like antagonist sulpiride (2 μM) and the D2R-like agonist quinpirole (10 nM, 100 nM, and 1 μM) to the slices while recording stimulus-evoked DA release. Notably, sulpiride did not impact DA release in response to single-pulse stimulation in either genotype (Figure S1A). In contrast, quinpirole reduced evoked DA release in a dose-dependent manner similarly in both genotypes (Figure S1B). These results indicate that the reduced DA release in eIF4E Tg mice is not dependent on altered D2R activity.

DA D1 receptors (D1Rs) are highly expressed by striatal projection neurons^63^ and interneurons^64^ and could indirectly contribute to DA release via circuitry-mediated effects. To test this possibility, we applied the DR1-like antagonist SCH23390 (1 μM) to the slices while recording stimulus-evoked DA release. Consistent with previous studies,^65,66^ we found that inhibition of D1Rs did not alter DA release in either genotype (Figure S1C).

Altogether, these results suggest that the reduced evoked DA release in the striatum of the eIF4E Tg mice is not attributable to defective DAT, D2R, and D1R functions.

### Unaltered DA metabolism and electrophysiological properties of midbrain DA neurons in the eIF4E Tg mice

Since the availability of DA for release is also regulated by the synthesis, degradation, and vesicular storage of DA within axonal terminals,^10^ we investigated whether alterations in these biochemical processes contributed to deficits in DA release observed in the eIF4E Tg mice. We employed western blotting to measure key proteins involved in DA synthesis (e.g., tyrosine hydroxylase [TH]), vesicular loading (i.e., vesicular monoamine transporter 2), and reuptake (i.e., DAT) in the striatum.^67,68^ Our analysis revealed that the levels of all the analyzed proteins were consistent between the eIF4E Tg mice and their WT littermates (Figures S1D and S1E). Immunostaining for TH also revealed a comparable density of midbrain DA neurons between genotypes (Figures S1F and S1G). Moreover, ultra-high-performance liquid chromatography-tandem mass spectrometry further established that the striatal levels of DA and its acidic metabolites 3,4-dihydroxyphenylacetic acid and homovanillic acid remained unaltered in the eIF4E Tg mice (Figure S1H).

While DA release is robustly controlled at the axonal level, the activity of the cell bodies is crucial for the overall regulation of DA neurotransmission.^10,69–73^ Therefore, we extended our investigation to determine whether overexpression of eIF4E alters the physiology of midbrain DA neurons.

Cell-attached recordings of DA neurons in acute midbrain slices^74,75^ demonstrated no significant differences in spontaneous firing frequency (Figures 2E and 2F) or coefficient of variation of the interspike interval (Figure 2G). Similarly, the whole-cell patch-clamp recordings revealed no differences in membrane (Figures 2H and 2I) and evoked firing (Figures 2L–2N) properties of DA neurons, aside from a modest increase in the voltage sag in response to hyperpolarizing currents (Figures 2J and 2K), suggesting a stronger activation of hyperpolarization-activated cyclic nucleotide-gated (HCN) channels.^74–76^ Finally, action potential frequency evoked by current injection (IF curves) was not different between genotypes (Figure 2N), indicating that DA neuron excitability remained unchanged in eIF4E Tg mice.

Altogether, these findings demonstrate that the eIF4E Tg mice exhibited reduced striatal DA release without significant DA neuron pathophysiology.

### Impaired DA release triggered by ACh interneurons in the eIF4E Tg mice

A growing body of evidence suggests that striatal DA release is modulated by local heterosynaptic regulatory mechanisms at the DA axons.^10,12,27,60^ Recently it has been established that synchronous activation of ACh interneurons and the subsequent ACh release can trigger heterosynaptic DA release through the activation of β2-nAChR on DA axons.^13,19,23,24^ Thus, we examined whether the reduced DA release in the eIF4E Tg mice originates from changes in heterosynaptic control of DA efflux by ACh.

**Figure 3.**
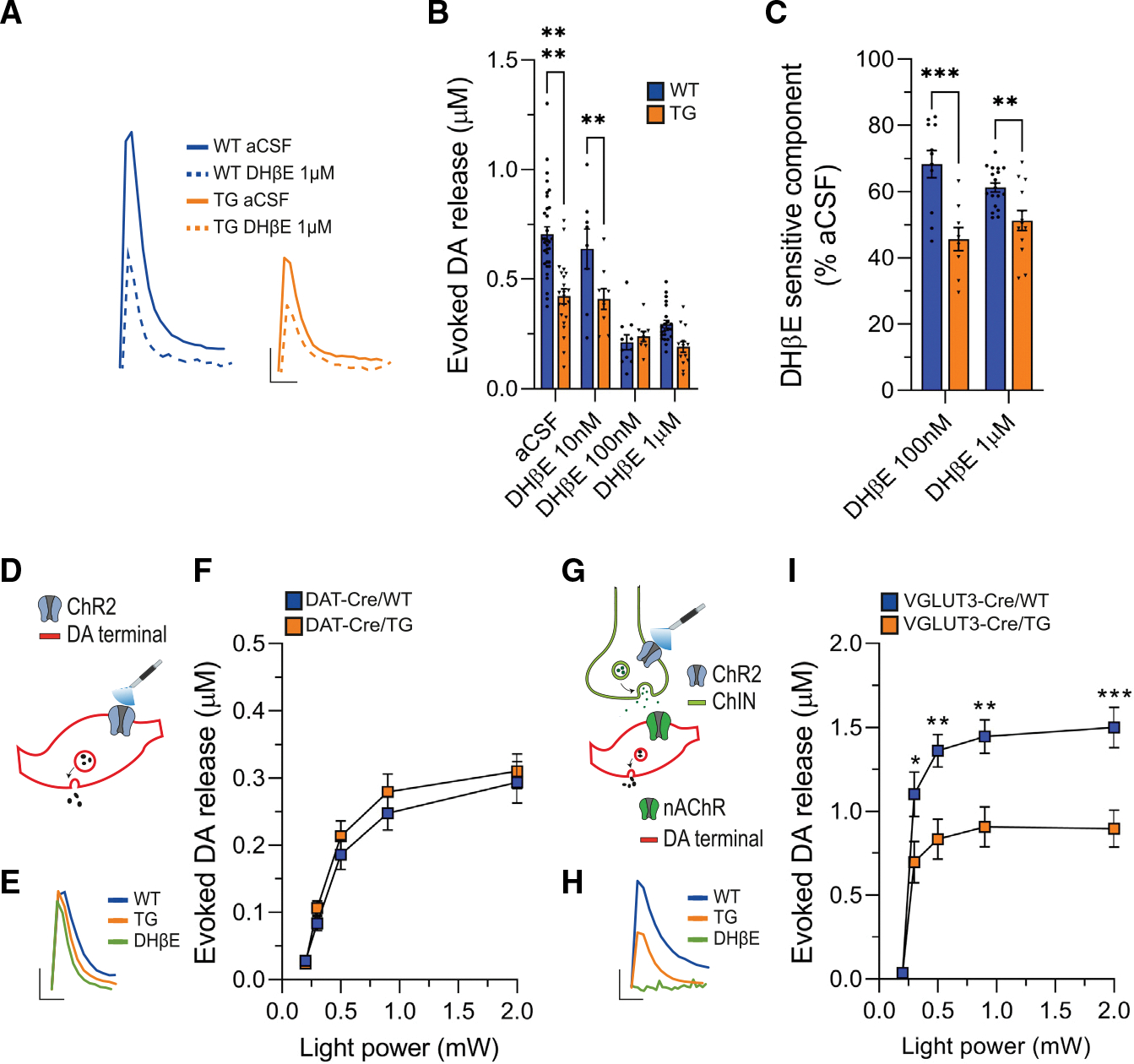
Impaired ACh-induced DA release in the eIF4E Tg mice (A) Representative FSCV traces of WT and eIF4E Tg (TG) before (artificial cerebrospinal fluid [aCSF]) and after DHβE (1 μM), a β2-nAChR inhibitor. Scale: 500 ms (x) and 0.1 μM (y). (B) Peak concentrations of DA release in DHβE (10 nM, 100 nM, and 1 μM) or aCSF after SP stimulus. Two-way RM ANOVA, genotype: F_(1, 39)_ = 26.19, ****p < 0.0001; treatment: F_(3,74)_ = 26.57, ****p < 0.0001; interaction genotype:treatment: F_(3, 39)_ = 8.09, ***p < 0.001; ****p < 0.0001 and **p < 0.01, Bonferroni’s multiple comparisons test. n = 8–18 slices/3–4 mice/treatment/genotype. (C) DHβE-sensitive DA release (% decrease from aCSF). Unpaired two-tailed t test, t_18_ = 4.089, ***p < 0.001 (100 nM DHβE), t_30_ = 3.363, **p < 0.01 (1 μM DHβE). (D and G) Experiment schematics. (E and H) Representative FSCV traces (scale: 500 ms [x] and 0.1 μM [y] [E] and 500 ms [x] and 0.2 μM [y] [H]). (F and I) Peak concentrations of DA release following photostimulation of (F) DA axons or (I) ACh interneuron at increasing light power (0.2, 0.3, 0.5, 0.9, and 2.0 mW). Two-way RM ANOVA for (F) genotype: F_(1, 24)_ = 0.5952; stimulation: F_(1.310, 31.44)_ = 162.4, ****p < 0.0001; interaction genotype:stimulation: F_(4, 96)_ = 0.6112, ns. n = 12–14 slices/4 mice/genotype; (I) genotype: F_(1, 17)_ = 9.814, **p = 0.0061; stimulation: F_(4, 68)_ = 96.00, ****p < 0.0001; interaction genotype: stimulation: F_(4, 68)_ = 5.924, ***p = 0.0004; *p < 0.05, **p < 0.01, ***p < 0.001, Bonferroni’s multiple-comparisons test. n = 12–14 slices/4 mice/genotype. Bars represent mean ± SEM, and dots represent individual slices. Statistics for non-significant data are shown in Table S3.

First, we performed FSCV in the presence of the β2-nAChR antagonist dihydro-β-erythroidine (DHβE; 10 nM, 100 nM, and 1 μM), which has been shown to significantly reduce electrically evoked DA release.^13,19,23,24^ As predicted, bath application of DHβE at concentrations of 100 nM and 1 μM reduced DA release triggered by a single-pulse stimulus in acute striatal slices obtained from WT mice (Figures 3A and 3B). In the eIF4E Tg mice, the effect of DHβE was attenuated (Figures 3A and 3B), and the DHβE-sensitive component of DA release was significantly smaller (Figure 3C).

To isolate the direct impact of altered DA release induced by ACh in eIF4E Tg mice, we selectively expressed the light-activated ion channel channelrhodopsin 2 (ChR2) in DA axons (Figures 3D–3F) or in ACh interneurons (Figures 3G–3I). For the selective targeting of ChR2 in DA axons, we bilaterally injected an adeno-associated virus (AAV) carrying Cre-inducible ChR2 into the substantia nigra of mice generated by crossing eIF4E Tg with DAT-Cre animals (DAT-Cre/WT and −/TG; STAR Methods).^77,78^ The selective expression of ChR2 in DA axons was confirmed by colocalization of mCherry expressed by AAV-ChR2 with TH via immunohistochemistry (Figure S2A).

For specific targeting of ChR2 in striatal ACh interneurons, we took advantage of their selective expression of VGLUT3.^79–85^ Accordingly, an AAV with Cre-inducible ChR2 was injected bilaterally into the striatum of eIF4E Tg and WT mice expressing Cre in VGLUT3-positive ACh interneurons (VGLUT3-Cre/WT and −/TG, respectively; STAR Methods).^86^ Selective expression of Cre in striatal ACh interneurons was confirmed by stereotaxic striatal injection of AAV carrying Cre-inducible yellow fluorescent protein (YFP) and immunostaining, revealing near-complete co-localization of the Cre-driven YFP with choline acetyltransferase (Figures S2B and S2C), a key enzyme for ACh synthesis in ACh interneurons.^79^

Next, we prepared acute striatal slices from DAT-Cre/WT and −/TG mice specifically expressing ChR2 in DA neurons. In this preparation, light stimulation selectively activates ChR2 on the axons of DA neurons, leading to transient DA release detected by FSCV (Figure 3D). Our findings indicate that optogenetically induced DA release, whether triggered by a single pulse at light power levels ranging from 0.2 to 2 mW (Figures 3E and 3F) or by train stimulation (Figure S2D), was not impaired in mice overexpressing eIF4E.

In contrast, in striatal slices from VGLUT3-Cre/WT and −/TG mice expressing ChR2 in ACh interneurons, light-evoked DA release requires the selective activation of ACh interneurons and functional β2-nAChR on DA axons^13,23^ (Figure 3G). Indeed, light-evoked DA release is completely abolished by the bath application of DHβE (1 μM; Figure 3H). Importantly, we observed impaired DA release triggered by ACh interneuron activation in eIF4E Tg mice, induced either by a single pulse with progressively increasing light power (Figure 3I) or by suprathreshold train stimulation (Figure S2E). These results suggest that the intrinsic mechanisms regulating axonal DA release in the eIF4E Tg mice are intact; the observed deficit in these mice is specifically attributed to compromised axonal DA release triggered by ACh.

### Unaltered firing properties, release, and total striatal content of ACh in the eIF4E Tg mice

**Figure 4.**
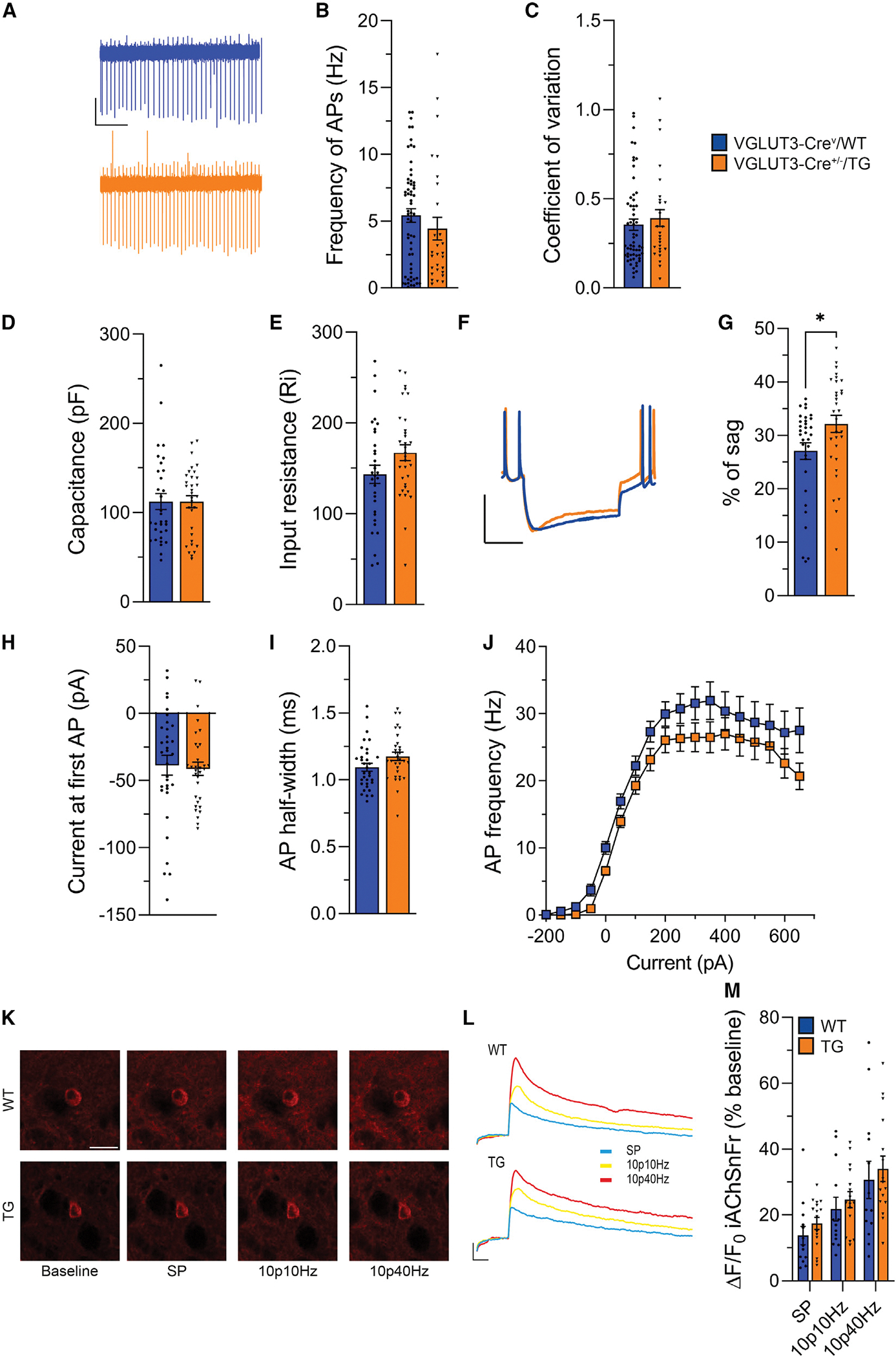
Preserved membrane properties of ACh interneurons and ACh release in the eIF4E Tg mice (A) Representative cell-attached traces. Scale: 1 s (x) and 50 mV (y). (B) AP frequency. Unpaired two-tailed t test: t_85_ = 0.88. (C) Coefficient of variation of interspike intervals. Unpaired two-tailed t test, t_85_ = 0.66, ns. (D) Membrane capacitance. Unpaired two-tailed t test: t_62_ = 0.01384, ns. (E) Input resistance. Unpaired two-tailed t test: t_62_ = 1.768, ns. (F) Representative voltage response to hyperpolarizing current injection (300 pA). Scale: 200 ms (x) and 50 mV (y). (G) Ratio of the instantaneous versus steady-state voltage response to −300 pA current injection (% of sag). Unpaired two-tailed t test: t_62_ = 2.280, *p = 0.0261. (H) Current at first AP. Unpaired two-tailed *t* test: t_63_ = 0.2984, ns. (I) AP half-width. Unpaired, two-tailed *t* test, t_43_ = 1.86, ns. (J) AP rate (Hz) in response to a series of current steps of increasing amplitude. Two-way RM ANOVA, genotype: F_(1, 63)_ = 3.820, ns; current step: F(_2.123, 133.7)_ = 97.00, ****p < 0.0001; interaction genotype:current step: F_(17, 1071)_ = 0.5234, ns. n = 28–59 neurons/4–9 mice/genotype. (K and L) Representative images (K; scale bar: 50 μm) and (L) iAChSnFr fluorescence at baseline and after SP, 10p10Hz, and 10p40Hz. Scale: 1 s (x) and 20% ΔF/F_0_ (y). (M) iAChSnFr fluorescence variation after SP, 10p10Hz, and 10p40Hz. Two-way RM ANOVA, genotype: F_(1, 27)_ = 0.5902, ns; stimulation: F_(1.036, 27.97)_ = 36.68, ****p < 0.0001; interaction genotype:stimulation: F_(2, 54)_ = 0.02027, ns. n = 13–16 slices/4 mice/genotype. Bars represent mean ± SEM, and dots represent individual neurons (A–J) or slices (K–M). Statistics for non-significant data are shown in Table S4.

To investigate whether the reduction in DA release triggered by ACh is linked to functional deficiencies in ACh interneurons of the eIF4E Tg mice, we conducted cell-attached (Figures 4A–4C) and whole-cell patch-clamp (Figures 4D–4J) recordings and examined tonic firing, membrane, and firing properties of ACh interneurons in acute striatal slices obtained from eIF4E Tg mice and WT littermates. To facilitate the identification of sparse striatal ACh interneurons, we employed VGLUT3-Cre/WT and −/TG mice injected with a Cre-dependent AAV expressing YFP (Figure S2B). This was complemented by their distinctive large cell somata and their ability to generate spontaneous action potentials, which is preserved in striatal slices.^87,88^ Our analysis revealed no significant differences in the spontaneous firing frequency (Figures 4A and 4B) or the coefficient of variation of the interspike interval (Figure 4C). Similarly, no differences were observed in the membrane (Figures 4D and 4E) and evoked firing (Figures 4H and 4I) properties of ACh interneurons, except for an increase in the voltage sag in response to hyperpolarizing currents (Figures 4F and 4G). Additionally, the IF curves were consistent between genotypes (Figure 4J), indicating that ACh interneuron excitability remained unaffected in the eIF4E Tg mice.

ACh release is inhibited by presynaptic muscarinic receptors.^89,90^ To determine whether altered inhibitory feedback contributes to the diminished DA release observed in eIF4E Tg mice, we measured electrically evoked DA release with FSCV in the presence of the muscarinic receptor agonist oxotremorine M (OxoM). Application of OxoM (10 μM) to striatal slices caused a significant reduction of evoked DA release in WT and eIF4E Tg mice (Figures S3A and S3B), indicating that the function of presynaptic muscarinic receptors is intact in the eIF4E Tg mice.

Our results so far could be attributed to a deficit in stimulus-evoked ACh release in eIF4E Tg mice. To explore this possibility, we first performed a colorimetric enzymatic assay to quantify ACh and choline concentrations in the striata dissected from the eIF4E Tg mice and WT controls.^91^ Our results showed no significant differences in normalized ACh and total choline concentration between the WT control and the eIF4E Tg mice (Figures S3C and S3D).

Next, we employed iAChSnFr, afluorescent genetically encoded ACh sensor, which provides a means for measuring synaptic ACh release in acute striatal slices with two-photon (2P) microscopy.^92^ An AAV carrying the iAChSnFr was injected into the striatum of the eIF4E Tg mice and WT littermates. Striatal slices prepared at least 3 weeks post surgery exhibited detectible iAChSnFr in the striatum, which responded to exogenously applied ACh with a dose-dependent increase in fluorescence (Figure S3E). Importantly, we observed no genotype-specific differences in the sensitivity of iAChSnFr for bath-applied ACh (50 μM) (Figures S3F and S3G).

We employed a series of electrical stimuli to induce ACh release in acute striatal slices and found no differences in the relative fluorescence of iAChSnFr between eIF4E Tg mice and their WT littermates at any of the stimulation patterns tested (Figures 4K–4M). These results indicate that ACh release elicited by electrical stimulation with frequencies similar to those used to elicit DA release in the FSCV experiments does not differ between eIF4E Tg and WT littermates.

Our results collectively demonstrate that the diminished DA release observed in the eIF4E Tg mice, while attributed to deficits in ACh signaling, is independent of ACh neurotransmission in the striatum.

### Impaired Ca^2+^ dynamics in the DA axons of the eIF4E Tg mice

Given the direct association between axonal Ca^2+^ levels and neurotransmitter release, we employed an independent measure of presynaptic function by selectively expressing the calcium sensor GCaMP6s (GCaMP) in the nigrostriatal terminals. To this end, we injected an AAV carrying the *Cre*-dependent GCaMP into the substantia nigra of DAT-Cre/WT and –/TG littermates.^88,93^ Three weeks later, we imaged fluorescent GCaMP transients in DA axons in acute striatal slices using 2P microscopy.

**Figure 5.**
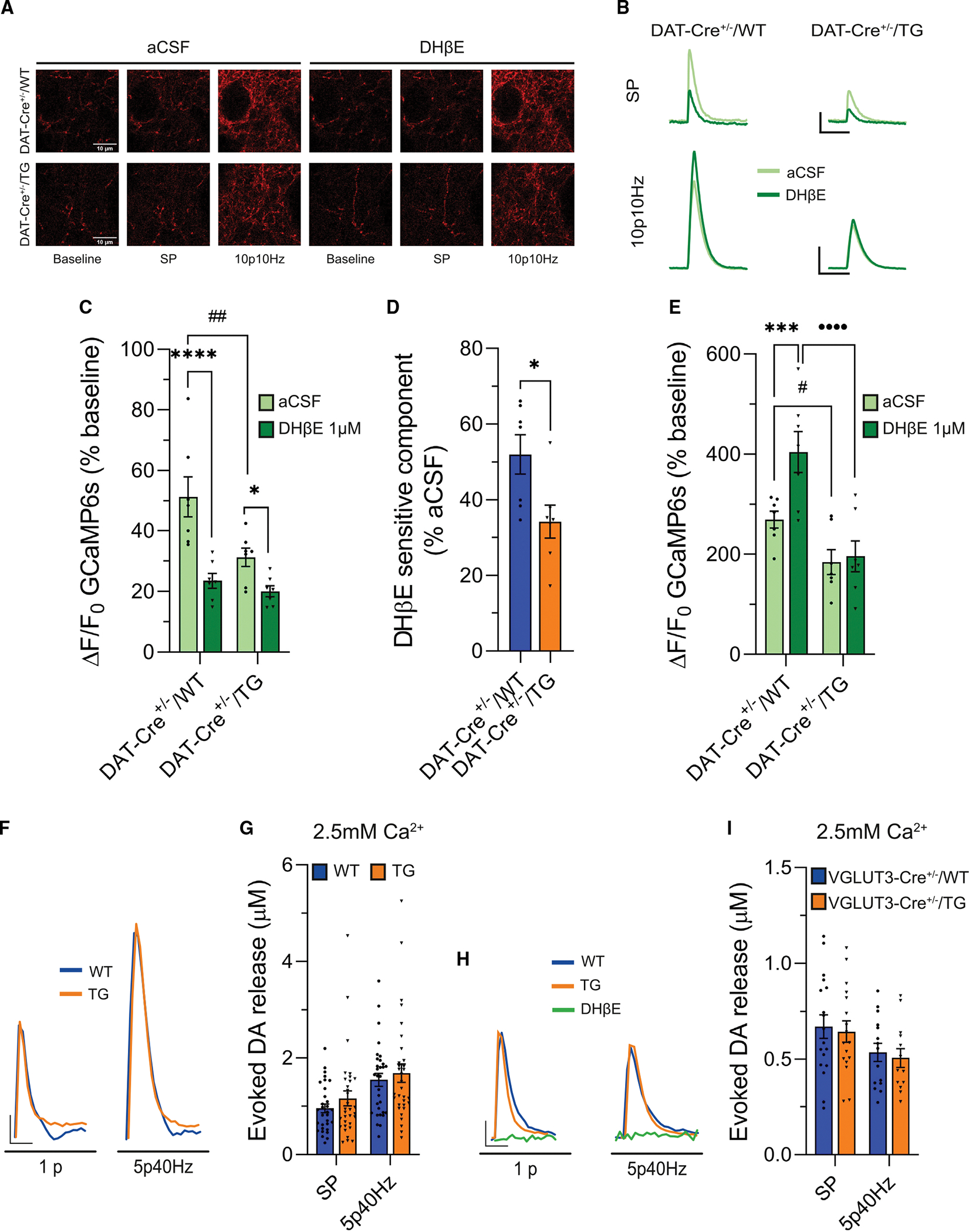
Altered presynaptic Ca^2+^ dynamics in DA axons and normalized DA released in high extracellular Ca^2+^ in the eIF4E Tg mice (A and B) Representative images (scale bar: 10 μm) and (B) GCaMP fluorescence traces in DA axons after SP or 10p10Hz in DHβE or vehicle (aCSF). Scale: 4 s (x) and 50% ΔF/F_0_ (y) for SP and 4 s (x) and 100% ΔF/F_0_ (y) for 10p10Hz. (C) Changes of GCaMP fluorescence after SP in DHβE or vehicle (aCSF). Two-way RM ANOVA, genotype: F_(1,12)_ = 6.426, *p = 0.0262; treatment: F_(1,12)=_43.43, ****p < 0.0001; interaction genotype:treatment: F_(1,12)_ = 7.745, *p = 0.0166; ****p < 0.0001, *p < 0.05 aCSF vs. DHβE for WT and TG, ##*p* < 0.01 WT vs. TG in aCSF, Bonferroni’s multiple-comparisons test. (D) DHβE-sensitive GCaMP fluorescence after SP. Unpaired two-tailed t test, t_12_ = 2.62, *p = 0.0224. (E) Changes in GCaMP fluorescence after 10p10Hz in DHβE or vehicle (aCSF). RM two-way ANOVA, genotype: F_(1,12)_ = 15.95, **p = 0.0018; treatment: F_(1,12)=_13.61, **p = 0.0031; interaction genotype:treatment: F_(1,12)_ = 9.57, **p = 0.0093; ***p < 0.001 aCSF vs. DHβE for WT, #*p* < 0.05 WT vs. TG in aCSF, ••••*p* < 0.0001 WT vs. TG in DHβE, Bonferroni’s multiple-comparisons test. n = 7 slices/3 mice/genotype. (F and G) Representative FSCV traces (F; scale: 500 ms [x] and 0.2 μM [y]) and (G) evoked DA release in 2.5 mM Ca^2+^ aCSF following SP and 5p40Hz. Two-way RM ANOVA, genotype: F_(1, 61)_ = 0.7065; stimulation: F_(1, 61)_ = 152,9, ****p < 0.0001; interaction genotype:stimulation: F_(1, 61)_ = 0.5063, ns. n = 31–32 slices/6–7 mice/genotype. (H and I) Representative FSCV traces (scale: 500 ms [x] and 0.1 μM [y]) and (I) evoked DA release induced by photostimulation of ACh interneurons in 2.5 mM Ca^2+^ aCSF following SP and 5p40Hz. Two-way RM ANOVA, genotype: F_(1,35)_ = 0.1698, ns; stimulation: F_(1,24)_ = 6.807, *p < 0.05; genotype:stimulation: F_(1,24)_ = 7.64, ns. n = 11–18 slices/3 mice/genotype. Bars represent mean ± SEM, and dots represent individual slices. Statistics for non-significant data are shown in Table S5.

Single-pulse stimulation elicited brighter axonal Ca^2+^ transients in slices from WT mice compared to the eIF4E Tg mice (Figures 5A–5C, aCSF, light green). To investigate the presynaptic role of β2-nAChR, we then repeated this protocol in the presence of DHβE (1 μM). In acute striatal slices from WT mice, bath application of DHβE reduced the brightness of the axonal Ca^2+^ transients, whereas this effect was attenuated in slices from the eIF4E Tg mice (Figures 5A–5C, DHβE, dark green). The blunted effect of DHβE on axonal Ca^2+^ influx of the eIF4E Tg mice is also indicated by a smaller DHβE-sensitive component (Figure 5D). Next, we tested the effect of train stimulation (10p10Hz) under the same conditions. Train stimulation elicited brighter axonal Ca^2+^ transients in slices from WT mice compared to the eIF4E Tg mice (Figures 5A, 5B, and 5E, aCSF, light green). However, reducing release probability by DHβE application only increased the relative axonal Ca^2+^ influx induced by train stimulation in WT slices (Figures 5A, 5B, and 5E, DHβE, dark green). By contrast, we did not observe a similar effect of DHβE in slices obtained from the eIF4E Tg mice (Figures 5A, 5B, and 5E, DHβE, dark green). Collectively, our data indicate that the eIF4E Tg mice exhibit altered heterosynaptic regulation of DAergic axons by ACh. This is evidenced by smaller activity-dependent axonal Ca^2+^ transients recorded using the GCaMP sensor expressed selectively in DA axons and characterized by attenuated DHβE sensitivity.

To explore this further, we manipulated the driving force for Ca^2+^ by increasing its extracellular concentration from 2 to 2.5 mM. We then conducted FSCV in acute striatal slices of eIF4E Tg and WT mice and induced DA release with electrical stimulation using a single pulse and a train (5p40Hz). In elevated extracellular Ca^2+^, we did not observe genotype differences in response to any of the stimuli tested (Figures 5F and 5G), indicating that higher extracellular Ca^2+^ levels compensate for the altered β2-nAChR-mediated release of the eIF4E Tg mice, leading to a normalization of DA release.

Finally, we investigated whether a similar increase in extracellular Ca^2+^ could normalize ACh-dependent DA release. We explored this possibility by measuring DA release with FSCV during optogenetic activation of ACh interneurons in striatal slices obtained from VGLUT3-Cre/WT and −/TG mice, as outlined in Figures 3G–3I. Under these experimental conditions, we observed an unaltered DA release in the eIF4E Tg mice (Figures 5H and 5I). Thus, higher levels of extracellular Ca^2+^ increase the release probability similarly in both genotypes, effectively bypassing the impaired β2-nAChR signaling in eIF4E Tg mice.

## DISCUSSION

Our study revealed reduced striatal DA release in the eIF4E Tg mice, associated with β2-nAChR dysfunction in DA axons. This dysfunction was unmasked by optogenetic stimulation of ACh neurons rather than direct DA terminal activation and occurred without affecting DA neuron morphology, biochemistry, firing activity, or ACh interneuron release and firing patterns. The reduced DA release correlated with behavioral inflexibility in the four-choice odor discrimination task, linking ASD symptomatology with striatal DA dysfunction.

The eIF4E Tg mice exhibited impaired reversal learning, consistent with other ASD models characterized by hyperactive mTOR signaling.^9,44,46,56,94^ For instance, a mouse model of Tuberous Sclerosis Complex (TSC) with *Tsc1* deletion in DA neurons (DA-TSC1 knockout [KO]) showed impaired reversal learning and reduced striatal DA.^56^ However, unlike the eIF4E Tg mice, DA-TSC1 KO mice have extensive morphological and physiological impairments in DA neurons,^56^ likely driven by hyperactive mTOR signaling, resulting in increased protein synthesis (via eIF4E) and reduced macroautophagy.^32,95,96^ Similar morphological changes are observed in mice with inactivated macroautophagy due to *Atg7* deletion in DA neurons.^97^ In contrast, eIF4E overexpression in our model primarily affects cap-dependent translation,^44–46^ leading to nuanced physiological changes in DA neuron function without overt morphological alterations. Yet, these changes suffice to induce behavioral inflexibility.

A similar pattern emerges in a mouse model of fragile X syndrome (FXS), characterized by increased eIF4E-dependent protein synthesis and hyperactive mTOR signaling.^98–100^ FXS mice also show reduced DA release^101^ and behavioral inflexibility,^102^ aligning with the deficits observed in eIF4E Tg mice.

We also observed a small but significant increase in membrane sag, a characteristic voltage response to hyperpolarizing current injections, in DA neurons and ACh interneurons of the eIF4E Tg mice without changes in passive membrane properties, spontaneous firing frequency, or action potential duration (Figures 2 and 4). The membrane sag increase mirrors that reported in CA1 hippocampal neurons of FXS mice, attributed to elevated expression of HCN channels,^103^ which are responsible for generating it.^104^ This shared feature suggests another physiological link between eIF4E Tg and FXS models besides the observed deficits in DA release.

Our data also demonstrate that the DA release deficits in the eIF4E Tg mice results from a malfunction of axonal β2-nAChR. Importantly, α4β2-subunit-containing nAChRs, which are expressed in DA axons, are reduced in postmortem brain tissues of patients with ASD, with no corresponding changes in mRNA levels, suggesting a post-transcriptional modification of these ion channel subunits.^105^ Mice lacking the β2 subunit of nAChRs display executive function deficits reminiscent of the perseverative behaviors observed in the eIF4E Tg mice and patients with ASD.^106,107^ Interestingly, these mice display reduced DA release at various stimulation frequencies while maintaining activity-dependent release dynamics.^25^ These findings align with our own results, showing that altered β2-nAChR function leads to a sustained reduction in DA release while maintaining activity-dependent release dynamics, similar to what is observed with eIF4E overexpression.

We further explored the presynaptic impairments of the eIF4E Tg mice by employing an independent measure based on the functional coupling between presynaptic Ca^2+^ levels and neurotransmitter release. To this end, we expressed GCaMP6 in DA axons and measured presynaptic Ca^2+^ transients in response to varying stimulation frequencies.^93^ β2-nAChR inhibition reduces the release probability at DA synapses, thereby facilitating release during high-frequency stimulation.^11,19,20,24^ Consistently, presynaptic Ca^2+^ transients showed facilitation in the presence of DHβE in WT mice (Figures 5A–5C), suggesting that β2-nAChR blockade reduces the release probability at DA synapses by altering presynaptic Ca^2+^ dynamics. The DHβE-induced facilitation of presynaptic Ca^2+^ transients, as well as the DHβE-sensitive component elicited by single-pulse stimulation, were both diminished in slices from the eIF4E Tg mice (Figures 5A–5D). Thus, our results obtained with presynaptic Ca^2+^ imaging and FSCV (Figure 3) reveal that the DA synapses of the eIF4E Tg mice maintain a high probability of DA release during β2-nAChR inhibition. In summary, these findings show a compromised high-pass filtering function provided by axonal β2-nAChRs on DA synaptic transmission in the eIF4E Tg mice.

Recent findings suggest that DHβE abolishes action potentials in DA axons induced by optogenetic activation of ACh interneurons, implying a direct role for β2-nAChRs in both axonal depolarization^23^ and release.^11,19,20,24,25^ These two effects could be interconnected yet independent, with depolarization depending on the influx of both Na^+^ and Ca^2+^ and the release relying solely on Ca^2+^. We note that our imaging and electrochemical recordings do not allow us to distinguish between these two effects of β2-nAChR function, and further studies are required to determine whether only release or both axonal action potentials and release are impaired in the eIF4E Tg mice.

In our study, we did not directly investigate the contribution of VGCCs to the presynaptic impairments observed in the eIF4E Tg mice. VGCCs, along with nAChRs, are critical for Ca^2+^ influx involved in axonal depolarization and DA release.^23,93,108–110^ A recent study has shown that genetic ablation of the N- and P/Q-type VGCCs, key players in DA transmission,^110^ significantly reduces DA release evoked by selective photostimulation of striatal axons and ACh interneurons.^23^ We cannot exclude the possibility that VGCCs downstream of nAChRs may contribute to the impaired DA release mediated by ACh (Figures 3I and S2E). However, the similar DA release observed in WT and eIF4E Tg mice following optogenetic stimulation of DA terminals (Figures 3F and S2D) suggests that the function of VGCCs remains unaltered in eIF4E Tg animals. This would be consistent with the preserved activity-dependent DA release dynamics observed in the eIF4E Tg mice (Figure 2D), which could also be affected by impaired VGCCs.

We also show that increasing extracellular Ca^2+^ levels rescue DA release evoked by electrical stimulation (Figures 5F and 5G) and optogenetic ACh release (Figures 5H and 5I). With elevated extracellular Ca^2+^, DA release induced by photostimulation of ACh interneurons remains dependent on β2-nAChRs, as it is abolished by DHβE application (Figure 5H). This suggests that increased Ca^2+^ levels can compensate for deficits in β2-nAChR function and restores DA release. However, the effect of elevated extracellular Ca^2+^ involves multiple mechanisms, including increased axonal depolarization, which requires further investigation in future studies.

While our data point to an altered function of β2-nAChRs in DA axons, the underlying molecular mechanisms remain speculative. nAChRs desensitize with kinetics influenced by their subunit composition.^111,112^ Given that eIF4E regulates the translation of specific “eIF4E-sensitive” mRNAs,^32,113–116^ it is conceivable that β2 subunits and/or other subunits expressed in DA terminals, such as α4 and α6,^11,21,117^ may be preferentially translated when eIF4E is overexpressed, leading to nAChRs with altered subunit composition and impaired desensitization dynamics. In line with this speculation, recent findings have demonstrated high expression of *CHRNB2*, which encodes the β2 subunits of nAChRs, in patient-derived induced pluripotent stem cells derived from individuals with FXS.^118^ Alternatively, altered nAChRs may be improperly trafficked or misplaced in DA axons. Future studies are necessary to elucidate how eIF4E overexpression affects the translatome of DA neurons, including the translation, assembly, and trafficking of nAChR subunits.

A compromised β2-nAChR high-pass filter, as revealed by our study, may offer a mechanistic explanation for understanding behavioral inflexibility in ASD. Indeed, β2-nAChR-triggered DA release is suggested to play a crucial role in amplifying physiologically relevant stimuli encoded by the various firing modes of DA neurons (i.e., tonic vs. burst firing).^10,26,68,73^ The aberrant β2-nAChR filtering system seen in the eIF4E Tg mice would not only hinder the dynamic probability of DA release in response to neuronal activity but would also lead to a desynchronization of ACh interneurons, since these interneurons respond directly to altered striatal DA levels via D2Rs.^26,119^

Behaviorally, the absence of dynamic amplification in DA levels and ACh desynchronization in response to salient stimuli may lead to an aberrant reward-prediction error, causing the eIF4E Tg mice to perseverate with an acquired behavioral strategy even if it is no longer reinforced.

Given the crucial significance of the coincident activity of DA and ACh neurotransmission in the striatum for the accurate computation of a reward-prediction error, pharmacological approaches aimed at indiscriminately elevating striatal DA levels might not adequately improve the cognitive aspects of repetitive and perseverative behaviors in ASD.

In conclusion, our study shows that, in the eIF4E overexpression model of ASD, striatal DA neurotransmission is hindered by aberrant functionality of β2-nAChRs on DA axons. These findings provide valuable insight into the intricate interplay between ACh and DA in striatum-dependent functions, highlighting the potential cellular origin of behavioral inflexibility present in ASD.

### Limitations of the study

Our study provides novel insights into the role of eIF4E overexpression in striatal DA signaling and behavioral flexibility in the context of ASD. However, several limitations should be acknowledged. First, while we demonstrated the involvement of impaired nAChRs in dysregulated DA release, we did not directly investigate other potential presynaptic mechanisms (e.g., VGCCs) that might also contribute to the observed deficits. Moreover, we did not explore whether compensatory mechanisms might develop over time in eIF4E Tg mice, which could impact their dopaminergic function and behavioral performance.

Second, our findings were based on acute slice preparations, which may not fully capture the *in vivo* dynamics of ACh-mediated DA release, especially under naturalistic conditions involving behavioral tasks. Finally, while we identified a specific role for β2-containing nAChRs in our study, the precise molecular mechanisms through which eIF4E overexpression alters nAChR functionality remain speculative and require further investigation.

These technical and conceptual limitations should be addressed in future studies to provide a more comprehensive understanding of the role of eIF4E overexpression in ASD.

### RESOURCE AVAILABILITY

#### Lead contact

Requests for further information and resources should be directed to and will be fulfilled by the lead contact, Emanuela Santini (emanuela.santini@ki.se).

#### Materials availability

This study did not generate new unique reagents.

#### Data and code availability

- All data reported in this paper will be shared by the lead contact upon request.
- This paper does not report original code.
- Any additional information required to reanalyze the data reported in this paper is available from the lead contact upon request.

## STAR★METHODS

### EXPERIMENTAL MODEL AND STUDY PARTICIPANT DETAILS

#### Animals

eIF4E^*wt/βtEif4e*^,^44,121^ VGLUT3-*Cre*^*wt*/+^ (Tg(Slc17a8-icre)1Edw/SealJ; JAX Strain#018147)^86^ and DAT-*Cre*^*wt*/+^ (Slc6a-Cre knockin)^77,78^ mice (referred to as eIF4E Tg, VGLUT3-*Cre* and DAT-*Cre*, respectively) were sex-separated and group-housed (up to 5 mice for cage) in a temperature (23C) and humidity (55%) controlled environment, on a 12-h light/dark cycle with water and food available *ad libitum*. Only male mice not older than 4 months were used for the experiments.^44^

All animal experiments were compliant with the ethical permit issued by the Swedish Board of Agriculture (Ethical number: 18194–2018 and 19345–2023) and were performed in accordance with the European Parliament and Council Directive 210&63/EU, 22nd September 2010 for experimentation animals’ protection.

#### Breeding strategy

The DAT-Cre, VGlut3-Cre and eIF4E Tg mice were maintained in hemizygosis by crossing them with C57BL/6J mice purchased from Janvier. Double transgenic mice were generated by crossing DAT-*Cre*^*wt*/+^ or VGLUT3-*Cre*^*wt*/+^ with eIF4E^*wt/βtEif4e*^. The offspring expressing *Cre* in DA neurons or striatal acetylcholine interneurons, respectively along with either overexpressing (DAT-*Cre*^*wt*/+^/eIF4E^*wt/βtEif4e*^ or VGlut3-*Cre*^*wt*/+^/eIF4E^*wt/βtEif4e*^ referred to as DAT-Cre/eIF4E and VGlut3-Cre/eIF4E, respectively) or not (DAT-*Cre*^*wt*/+^/eIF4E^*wt/wt*^ or VGlut3-*Cre*^*wt*/+^/eIF4E^*wt/wt*^ and referred to as DAT-Cre/WT and VGLUT3-Cre/WT) eIF4E were utilized in this study. All mice were maintained in a C57BL/6J background.

#### Genotyping

Genotyping was done by PCR; genomic DNA was obtained from earmarking biopsies. Tissue samples were digested overnight (minimum 6h) at 56°C, shaking at 1000–1100 rpm, in 400 μL of the following buffer: 100 mM Tris-HCL pH 7.5, 1 mM EDTA, 250 mM NaCL, 0.2% SDS, Proteinase K (Invitrogen) 0.1 mg/mL. Afterward, lysates were diluted 1:10 in dH2O and used as template for PCR reaction carried out using the KAPA2G mastermix (Merck). Primers (Thermo Fisher Scientific) used in eIF4E transgene amplification were: 5′-CACAGCTACAAAGAGCGGCTCCACC −3′ and 5′-CACTGCATTCTAGTTGTGGTTTGTCC-3’. Those used for DAT-Cre amplification were: 5′-CATGGAATTTCAGGTGCTTGG-3′, 5′-ATGAGGGTGGAGTTGGTCAG-3′, 5′-CGCGAACATCTTCAGGTTCT-3′, and for vGLUT3-Cre amplification: 5′-ACACCGGCCTTATTCCAA G-3′, 5′-AGATGTCTTATGGAGCCACCAC-3′, and 5′-CTGAGACCAAGGTCCATATTCC-3’.

### METHOD DETAILS

#### Acute brain slices

For FSCV experiments, brain slices were prepared as follow^123^: mice underwent cervical dislocation, and the brain was removed and placed on a high sucrose cutting solution, previously cooled to 4°C: 10 mM NaCl, 2.5 mM KCl, 25 mM NaHCO_3_, 0.5 mM CaCl_2_,7 mM MgCl_2_, 1.25 mM NaH_2_PO_4_, 180 mM sucrose, 10 mM glucose; bubbled with 95% O_2_/5% CO_2_ to pH 7′4. Brains were mounted on a VT1200 vibratome (Leica Biosystems) and coronal sections (250 μm) including the striatum were collected. Slices were transferred to Artificial Cerebro-spinal Fluid (aCSF) containing (in mM): 125 NaCl, 2.5 KCl, 25 NaHCO_3_, 2 CaCl_2_, 1 MgCl_2_, 1.25 NaH_2_PO_4_, and 10 glucose bubbled with 95% O_2_/5% CO_2_ to pH 7.4, at 34°C for 30 min. After that, the slices were kept in oxygenated (95% O_2_/5% CO_2_) aCSF at room temperature.

For Patch-Clamp electrophysiology, and 2P Microscopy acute brain slices were prepared and maintained using the two-step *N-*methyl-D-glucamine (NMDG) based protective recovery method.^124^ Slices were initially prepared using NMDG-based solution containing (in mM): 92 NMDG, 30 NaHCO_3_, 2.5 KCl, 20 HEPES, 2 thiourea, 1.25 NaH_2_PO_4_, 3 Na-pyruvate, 5 ascorbic acid, 25 glucose, 0.5 CaCl_2_ and 10 MgCl_2_ titrated to pH 7.4. During the experiment, the slices were stored in the HEPES-based holding solution containing (in mM): 92 NaCl, 30 NaHCO_3_, 2.5 KCl, 20 HEPES, 2 thiourea, 1.25 NaH_2_PO_4_, 3 Na-pyruvate, 5 ascorbic acid, 25 glucose, 2.5 CaCl_2_ and 2 MgCl_2_ titrated to pH7.4. Mice underwent cardiac perfusion with 25 mL chilled NMDG solution prior to brain removal and dissection. Brains were mounted on a VT1200 vibratome (Leica Biosystems) and coronal sections (250 μm) containing the striatum or the substantia nigra were collected and stored in NMDG-based solution for 10 min at 32°C. Subsequently, the slices were maintained in HEPES-based holding solution at room temperature. All solutions were oxygenated with 95% O_2_ and 5% CO_2_.

#### Fast-Scan Cyclic Voltammetry (FSCV)

Carbon fiber microelectrodes (CFMs) were made in house as follows: 7μm thick carbon fibers (Good Fellow, Huntingdon, GB) were inserted into 0.69 × 1.20 × 100 mm borosilicate glass tubes (Science Products). Tubes were pulled to seal the glass around the fiber, and the exposed fiber was cut ~150 μm length. To improve the seal around the carbon fiber, CFMs tips were immersed in melted paraffin, and extra coating was removed with xylene.^125^ CFMs were calibrated after each recording by quantifying DA at known concentrations (1.25, 2.5, 5 μM).^126^

Striatal slices were placed in the submersion recording chamber of the rig and continuously perfused with oxygenated (95% O_2_/5% CO_2_) aCSF at 32°C–34°C for at least 10 min before starting the recordings. CFMs filled with 1M KCl, were inserted in the *dorsal* striatum and received a triangular voltage wave (−0.4V to +1.2V at 400V/s) every 100 ms. The resulting currents were measured with a Chem-clamp 5MEG amplifier (Dagan corporation, Minnesota, USA). Slices were stimulated with a bipolar stainless-steel electrode placed ~150μm from the recording electrode. Using an Iso-Flex stimulus isolator (A.M.P.I.), single pulses (1 ms × 300 μA) were applied every 90s to determine a stable DA release. For experiments involving train stimulations, 250s were allowed between stimuli. Recordings and data quantification were done with the Demon Voltammetry suite software.^61^

All drug inhibitors were dissolved in water, unless otherwise stated and bath applied for at least 15 min after achieving a stable baseline. We employed the following drugs: DHβE (Dihydro-β-erythroidine hydrobromide, Tocris) (10 nM, 100 nM, 1 μM), 10 μM Oxotremorine (Tocris), 2 μM Sulpiride (Sigma; dissolved in DMSO), SCH23390 1 μM (Tocris; dissolved in DMSO), Quinpirole (10 nM, 100 nM, 1 μM) (Tocris). The concentrations employed in these studies are in a range compatible with previously published FSCV experiments.^13,24,89^

When modified aCSF was used, cations concentrations were adjusted to maintain osmolarity: from 2 mM Ca^2+^/1 mM Mg^2+,^ to 2.5 mM Ca^2+/^0.5 mM Mg^2+^.

In FSCV recordings with optogenetic stimulation, optical probe was placed ~150μm from the recording electrode, and 470nm light (at different light intensities: 0.2, 0.3, 0.5, 0.9, 2 mW) was delivered in 3ms pulses every 120s.

#### Patch-clamp electrophysiology

Recordings were obtained using a patch clamp electrophysiology rig fitted with SciCam pro camera (Scientifica, United Kingdom) equipped with a 40 ×0.8 NA water-immersion objective (LUMPlanFLN, Olympus, United States) and Dodt contrast tube optics. Recordings were obtained with a Multiclamp 700B amplifier (Molecular Devices) and Axon Digidata 1550B digitizer (Molecular Devices, United States), using pCLAMP 11 software (Molecular Devices, United States). Data were low-pass filtered at 10 kHz. For all recordings, borosilicate glass capillaries were used to prepare electrodes with 3–4MOhm tip resistance. Slices were maintained in a submersion recording chamber with oxygenated (95% O2/5% CO2) aCSF supplied at 3 mL/min and maintained at 32°C–34°C.

##### Intracellular and extracellular recordings, identification of DA neurons and ACh interneurons

Recording electrodes were filled with potassium-gluconate based internal solution containing (in mM): 120 K-gluconate, 20 KCl, 4 MgATP, 0.3 Na2GTP, 5 Na2-phosphocreatine, 0.1 EGTA and 10 HEPES at pH 7.25 and osmolarity ~300 mOsm. Neurobiotin (Vector Labs, 0.2%) was added to the internal solution for post-hoc identification of DA neurons.

Prior to breaking through the membrane, cells were held in cell-attached configuration with voltage held at −70 mV for 1 min to measure extracellular firing. The rest of the recordings taken upon entering whole-cell configuration were performed in current-clamp mode. Ramp current injection was performed ranging from −300 to +700 pA over 1 s with 1 s intersweep interval. Following that we performed stepwise current injection from −300 pA to +700 pA in 50 pA increments. Each current step was held for 500 ms with 500 ms intersweep interval. Access resistance (Ra) was measured before and after the current injection recordings in voltage-clamp mode and recordings were discarded if Ra exceeded 30 MOhm or changed by over 10%.

Dopaminergic cells were initially identified based on size as well as firing properties upon entering whole-cell configuration. After recording, midbrain slices were fixed overnight in 4% paraformaldehyde (PFA) in 0.1 M PBS (pH 7.4) at 4°C and underwent immunofluorescence staining to identify biotin-filled DA neurons.^123^ Briefly, following a series of washes in tris-buffered saline (TBS), slices were incubated in Streptavidin Alexa Fluor-647 (Invitrogen, 1:200) in 0.6% Triton X-100 in TBS for 48 h. Slices were then incubated for 1 h in blocking solution containing 10% normal goat serum (NGS) in 0.6% TBS-TritonX100 followed by immunostaining for tyrosine hydroxylase (TH) which was used as a cellular marker for dopaminergic cells. Cells were incubated in 2% NGS/0.6% TBS-Triton containing mouse anti-TH (Millipore, #MAB318, 1:1000) for 72 h, followed by overnight incubation in 2% NGS/0.6% TBS-triton with goat anti-mouse Alexa Fluor Plus 488 (Invitrogen, #A-32723, 1:500). Only recordings obtained from neurons positive for TH, and thus considered DA neurons, were included in this study.

Identification of ACh interneurons was facilitated by injecting VGLUT3-Cre/WT and -TG mice in the striatum with an AAV5 expressing EYFP in a Cre-dependent manner, aiding in the visualization of cholinergic interneurons. Due to the fluorescent labeling, coupled with the large soma size and tonic activity of cholinergic interneurons, the addition of biotin to the internal solution was not necessary, and post-hoc immunohistochemical identification of the recorded neurons was not performed.

##### Analysis of patch-clamp recordings

Analysis of patch-clamp data was performed using either ClampFit (Molecular Devices) or the open-access Python-based software Stimfit 0.15.^122^ The extracellular firing frequency of DA and ACh neurons was determined by counting spikes in a 60 s or 10 s window, respectively, to determine the rate per second. The coefficient of variation was calculated by dividing the standard deviation by the mean of the interspike interval (VC = σ/μ), for each experimental group.

The current at which the first action potential fired with ramp current injection was used to determine the rheobase current. Action potential duration was determined using the half-width of the action potential, which measures the difference in time between the half-max amplitude during depolarisation and repolarisation. Current-frequency (IF) plots were generated from the action potential frequency (Hz) at each current step. Sag ratio was determined using the ratio between the steady state decrease in voltage and the peak decrease in voltage with the hyperpolarising current injection of −300 pA. The voltage response to −100 pA current injection was used to determine the input resistance (Ri) and an exponential line fit from the initial voltage change was used to determine the membrane time constant (τ). Membrane capacitance was calculated using τ/Ri.

#### 2 photon microscopy

Acute striatal slices were transferred to a recording chamber and allowed to stabilize for at least 15 min. After, a tungsten concentric bipolar electrode with a 3 μm tip size (World Precision Instruments) was positioned on the surface of the slice, and images were captured approximately ~150 μm away from the stimulating electrode. Using two IsoFlex stimulus isolators (A.M.P.I.) connected in parallel, slices were stimulated with the following protocol (±250μA × 500 μs): a single pulse, followed by 10 pulses at 10Hz, and another set of 10 pulses at 40Hz, with a 250 s interval between stimulations. Fluorescent biosensors were excited with a Ti:sapphire laser (Ultra II, Coherent) and imaging was performed using a commercially available 2-photon microscopy setup (Scientifica) equipped with a 16× objective, 0.8 NA (Nikon).

Axonal-GCaMP6s and iAChSnFr were excited at 920 nm and 950 nm respectively. Images were acquired with the following settings: 5x zoom, 512 × 512 px frame size, 1 frame/image (scan rate 1Hz).

Image quantification was performed using ImageJ, where fluorescence over time was analyzed. The baseline for fluorescence values was established by averaging frames before stimulation. Subsequently, maximal fluorescence values were normalized to the calculated baseline, and changes in fluorescence were quantified through baseline subtraction.

#### Western blot

Striatal sample preparation was performed as follows: mice were rapidly decapitated, and their head was briefly placed in liquid nitrogen.^127^ Brains were subsequently removed and the striata were rapidly dissected and flash frozen in liquid nitrogen. The tissues were sonicated in 750 μL of 1% SDS and the homogenates were boiled at 100°C for 10 min. The protein content of the homogenates was determined with the Pierce BCA Protein Assay kit. The samples were prepared by adding Lemli buffer (4X) and by boiling the samples for another minute. Equivalent amount of protein per sample (25–30 μg/well) were loaded into 10% polyacrylamide gels.^127^ Proteins were transferred to Immobilon FL PVDF membranes (pore size 0.2 μm). Blots were blocked with a blocking solution composed of Odyssey Blocking Buffer: TBS +0.1% Tween 20 (TBST) in a ratio 1:1 for 1 h at room temperature. Blots were then incubated with primary antibody (Cell Signaling: DARPP-32, #: 2306S and actin, # 4970; Millipore: TH, #MAB318 and DAT, #MAB369; Sigma-Aldrich: vMAT2, #V9014); all diluted 1:1000) diluted in Odyssey Blocking Buffer overnight at 4°C. Blots were then washed with TBST and incubated with secondary antibodies (IRDye, LI-COR Biosciences: anti-rabbit, #925–68071 for DARPP-32, actin and vMAT2; and anti-mouse, #926–32210 for TH; diluted 1:10000) for 1 h at room temperature. Blots were developed using the Odyssey imaging system (LICOR). Western blots were analyzed in Image Studio Lite (LICOR).

#### Ultra-high performance liquid chromatography-tandem mass spectrometry (UHPLC-MS/MS)

Concentrations of DA, DOPAC and HVA in the striatal samples were measured by UHPLC-MS/MS following derivatization with benzoyl chloride.^128,129^ Briefly, 20 μL volumes of the deproteinated supernatants from the tissue homogenates (1:10 w/v in MeOH) were mixed with 20 μL of the internal standard mixture (1 μM of each deuterated analyte standard DA-d4, DOPAC-d5, HVA-d3 in MeOH) followed with pipetting 20 μL benzoyl chloride (2% in ACN) and 20 μL sodium carbonate (100 mM in water, pH 10.5). The mixtures were vigorously shaken after each pipetting step. The resulting derivatized solutions were mixed for 5 min. The reaction was terminated by pipetting 20 μL sulfuric acid (1% in water), 10 μL of the final solution was injected on column. The UHPLC-MS/MS system included a Waters Xevo TQ-S micro triple quadrupole mass spectrometer with the electrospray ionization source operating in a positive mode, and an ACQUITY UPLC system (all purchased from Waters Corporation, Milford, MA, USA). The calibration curves were constructed in the range of 0.2–1000 ng/mL.

#### Detection of striatal ACh content

ACh levels in the striatum were measured with Choline/Acetylcholine Assay kit (Abcam, #ab65345) following the colorimetric procedure.^91^ Briefly, mice were rapidly decapitated, and their head was briefly placed in liquid nitrogen. Brains were removed and the striata were rapidly dissected and flash frozen in liquid nitrogen. Each striatum was homogenized in 50 μL of ice-cold Choline Assay Buffer with a Dounce homogenizer. The homogenates were transferred to new tubes, centrifugated at 14,000 rpm for 10 min at 4°C. An aliquot (10 μL) of each sample was transferred to a new tube and utilized to measure the protein content with Pierce BCA Protein Assay kit. The remaining samples were subjected to the Choline/Acetylcholine assay as per manufacture’s instruction.

#### Immunohistochemistry

Animals were deeply anesthetized with Pentobarbital, and transcardially perfused with 0.9% saline solution for 1 min followed by 4% paraformaldehyde (PFA) in phosphate buffer for 10 min. Brains were extracted and post-fixed overnight (same paraformaldehyde solution), cut in 30 μm thick sections using a VT1200 vibratome (Leica Biosystems) and stored in cryoprotectant solution (30% Glycerol, 30% Ethylene glycol in 0.1 M NaHPO_4_) at −20°C. Brain sections were processed for immunohistochemistry as follows: sections were washed three times in PBS for 10 min, permeabilized in 0.1% Triton X-100 in PBS (PBS-T), three times for 10 min, and incubated for 1 h in blocking solution containing: 2% Normal Goat Serum (NGS), and 0.1% PBS-T. After blocking, sections were incubated overnight at 4°C in 2% NGS +0.1% PBS-T containing a combination of the following antibodies: guinea pig anti-ChAT (Synaptic Systems, #297015, 1:500), mouse anti-Tyrosine Hidroxylase (Millipore, #MAB318, 1:100), chicken anti-GFP (Abcam, #Ab13970, 1:2000). After washing, sections were incubated for 2 h with 2%NGS and 0.1%PBS-T containing a combination of the following secondary antibodies (Thermo Fisher Scientific, for all 1:500): goat anti-rabbit conjugated with Alexa 488 (#A-11034), goat anti-chicken IgY conjugated with Alexa 488 (#A-11039), goat anti-guineapig conjugated with Alexa 568 (#A-11075). Sections were mounted, after washing, using a medium for fluorescent sections: Fluromount-G with DAPI (Invitrogen).

For Immunofluorescence quantification, sections were imaged at 20x with confocal microscope (Zeiss LSM800). To avoid biases in quantification, z-stacks at 1μm intervals across the vertical axis of the section were acquired. Afterward, images were processed with ImageJ to compile the z-stacks into single images, and immunofluorescent cells were manually counted.

#### Stereotactic surgeries

Mice were injected with buprenorfine (intraperitoneally (i.p.); 5 mg/kg), anaesthesized with isoflurane (2%) and placed in a stereotaxic frame (Stoelting). Adenovirus (AAV) injection was performed using a glass pipette (Drummond, 15–20 μm tip size). A fitted plunger controlled by a hydraulic injector (Quintessential Stereotaxic Injector, Stoelting), was inserted into the pipette, and used to inject the viral solutions (250 nL and 100 nL in striatum and SNc, respectively) at the steady rate of 50 nL/min. We utilized the following coordinates (from bregma): Striatum (0.8 AP, 1.8 ML, 2.4 DV), SNc: (−3.1 AP, 1.25 ML, 3 DV). Rimadyl and buprenorphine (i.p., 0.1 mg/kg) were administered twice a day for 3 days after surgery. The mice were left to recovery after surgeries for at least three weeks before being employed in other type of experiments.

AAVs injected in the striatum: pAAV-Ef1a-DIO EYFP was a gift from Karl Deisseroth (Addgene viral prep #27056-AAV5; http://n2t.net/addgene:27056; RRID:Addgene_27056), pAAV.hSynap.iAChSnFR was a gift from Loren Looger (Addgene viral prep #137950-AAV1; http://n2t.net/addgene:137950; RRID:Addgene_137950),^92^ pAAV-EF1a-DIO-hChR2(H134R)-mCherry-WPRE-HGHpA was a gift from Karl Deisseroth (Addgene viral prep #20297-AAV5; http://n2t.net/addgene:20297; RRID:Addgene_20297). Those in the SNc: pAAV-hSynapsin1-FLEx-axon-GCaMP6s was a gift from Lin Tian (Addgene viral prep #112010-AAV5; http://n2t.net/addgene:112010; RRID: Addgene_112010),^120^ pAAV-EF1a-DIO-hChR2(H134R)-mCherry-WPRE-HGHpA was a gift from Karl Deisseroth (Addgene viral prep #20297-AAV5; http://n2t.net/addgene:20297; RRID:Addgene_20297).

#### Four-choice odor discrimination and reversal-learning paradigm

Adult male mice were subjected to the four-choice odor discrimination and reversal-learning paradigm^53–56^ to assess learning and cognitive flexibility. The mice were food restricted with *ad libitum* access to drinking water and maintained at around 80% of their body weight until completion of the behavioral tests. The food restriction began 3 days before pre-training. The animal weights were recorded in the morning and food was given at the end of the day.

We found no significant differences in the body weight of wild-type and eIF4E Tg mice prior the test (WT: 30.8 ± 1.1 g and TG: 29.4 ± 0.9 g), during the three days of food restriction (e.g., weight at the last day of food restriction WT: 27.2 ± 0.9 g and TG: 25.9 ± 0.9 g) and the three days of the test (e.g., weight at the last day of the test WT: 25.2 ± 0.8 g and TG: 24.1 ± 0.6 g) as indicated by Two-way RM ANOVA, showing a main significant effect of the Time (F_(6, 72)_ = 91.5, *p* < 0.0001) but not main significant effect of the genotype (F_(1, 12)_ = 1.1, *p* = 0.3) and Time:Genotype interaction (F_(6, 72)_ = 0.2, *p* = 0.9).

The test arena consisted in an opaque white custom-made box (45 × 45 × 45 cm). Odor stimuli were presented in dark ceramic pots (measuring 7cm in diameter and 5cm in depth) located in each corner of the box. Pots were sham baited with Cheerios Honey cereals (Nestle’ Sverige AB, Helsingborg, Sweden) placed underneath wood shavings. The apparatus and the pots were cleaned with 70% ethanol and carefully washed with soap at the end of each testing day.

The first day of pre-training (day1; see schematic Figure 1A), mice were allowed to freely explore the testing arena and the pots. Around 1/8^th^ of Cheerios Honey was placed inside each empty pots, located in the four corners of the box. The mouse was placed in a central glass cylinder that was lifted to allow exploration and consumption of the cereals in the pots. After 10 min, the mouse was returned in the cylinder and the pots re-baited. This was repeated 3 times for a total habituation time of 30 min.

The second pre-training day was the shaping phase of the test (day 2) used to teach the mice to dig the food rewards (1/8^th^ of Cheerios Honey) buried in wood shavings. In the shaping phase, only one pot was employed with increasing amount of shaving material to gradually cover the food reward. The corner containing the pot was alternate in each trail and all corners were equally rewarded. Trials were untimed and consisted of 2 trials without wood shavings, two trials with a dusting of shaving, two trails with a quarter full pot, two trails with half full pot and four trials with the food reward completely buried.

On the odor discrimination and reversal tests (day 3) the wood shavings were freshly scented with organic essential oils (Örtagubben AB, Stockholm, Sweden). All the essential oils (100% pure) were diluted 1:10 in odorless mineral oil and mixed at 0.05 mL/g of shavings. In both tests, the stimulus presentation was pseudo-randomized such that an odor was never in the same quadrant two trials in a row. In the discrimination and reversal tests *criterion* was met when the animal completed 8 out of 10 consecutive trials correctly.

During the discrimination phase the mice had to discriminate between four different odors (O1–4: thyme, clove, rosemary, and cardamom) and learn the one (O1: thyme) associated with a buried food reward (1/8^th^ of Honey Nut Cheerio). Each trial began with the mouse confined in the central starting cylinder equidistant to the four odor pots. Timing started when the cylinder was lifted, and the mouse was free to explore the arena until it chose to dig in one of the pots. Digging was defined by purposefully moving the shavings with the paws. If an incorrect choice was made and/or prevent multiple digging choice, the mouse was cornered with a grid and the trial was considered terminated. A trial was also terminated if no choice was made within 3 min from the lifting of the starting cylinder and it was recorded as an *omission*. If the animal had two omission trials in a row, digging was reinstated by placing a pot of unscented shavings with a well exposed food reward at the center of the arena. All pots were removed from the maze and rebaited, if necessary.

Once criterion was met in the discrimination phase the mice were moved to the reversal phase. All shavings were replaced with new shavings to prevent discrimination via unintentional cues. O4 (cardamom) was swapped for a novel odor (O5: eucalyptus) and in this phase the rewarded odor was O2 (clove). *Perseverative errors* were defined as choices to dig in the pot with the odor rewarded during the discrimination phase (O1: thyme). *Regressive errors* were choices to dig in the pot rewarded in the discrimination phase (O1: thyme; i.e., perseverative errors) made after a correct choice (i.e., retrieval of food reward in pot with O2: clove). *Irrelevant errors* were choices to dig in the pot with the odor that was never rewarded (O3: rosemary). *Novel errors* were choices to dig in the pot with the newly introduced odor (O5: eucalyptus), which was also never rewarded. *Omissions* were trials where no digging choice was made within 3 min from the start. Total errors are the sum of perseverative, regressive, irrelevant, novel and omission errors.

### QUANTIFICATION AND STATISTICAL ANALYSIS

All data are presented as mean ± SEM. Statistical analyses were performed using Prism 10 (GraphPad). The statistical tests applied for comparisons include Student’s paired or unpaired two-tailed t test, as well as two-way or two-way repeated measures (RM) ANOVA, where appropriate. Detailed statistical information, including the specific test used, exact *N* values (indicating number of slices, samples or neurons/animals as relevant), and descriptive statistic can be found in the figure legends and Tables S1–S8. The statistical significance was defined as *p < 0.05, **p < 0.01, ***p < 0.001 and ****p < 0.0001.

## Supplementary Material

NIHMS2124379 Supplemental Material

SUPPLEMENTAL INFORMATION

Supplemental information can be found online at https://doi.org/10.1016/j.celrep.2024.114997.

## Figures and Tables

**KEY RESOURCES TABLE:** 

REAGENT or RESOURCE	SOURCE	IDENTIFIER

Antibodies		
DARPP-32	Cell Signaling	Cat#: 2306; RRID:AB_823479
actin	Cell Signaling	Cat# 4970; RRID:AB_2223172
TH	Millipore	Cat#: MAB318; RRID:AB_2201528
DAT	Millipore	Cat#: MAB369; RRID:AB_2190413
vMAT2	Sigma-Aldrich	Cat#: V9014; RRID:AB_1841254
IRDye 800CW goat anti-mouse IgG	LI-COR Biosciences	Cat#: 926-32210; RRID:AB_621842
IRDye 800CW goat anti-rabbit IgG	LI-COR Biosciences	Cat#: 925-68071; RRID:AB_2721181
ChAT	Synaptic Systems	Cat#: 297015; RRID:AB_2744644
GFP	Abcam	Cat#: ab13970; RRID:AB_300798
Goat anti-rabbit IgG Alexa Fluor 488	Thermo Fisher Scientific	Cat#: A-11034; RRID:AB_2576217
Goat anti-chicken IgY Alexa Fluor 488	Thermo Fisher Scientific	Cat#: A-11039; RRID:AB_2534096
Goat anti-guinea pig IgG Alexa Fluor 568	Thermo Fisher Scientific	Cat#: A-11075; RRID:AB_2534119
Goat anti-mouse IgG Alexa Fluor Plus 488	Thermo Fisher Scientific	Cat#: A-32723; RRID:AB_2633275
Bacterial and virus strains		
pAAV-Ef1a-DIO EYFP	It was a gift from Dr. Karl Deisseroth	Cat#: 27056-AAV5; RRID:Addgene_27056
pAAV.hSynap.iAChSnFR	Borden et al.^92^	Cat#: 137950-AAV1; RRID:Addgene_137950
pAAV-EF1a-DIO-hChR2(H134R)-mCherry-WPRE-HGHpA	It was a gift from Dr. Karl Deisseroth	Cat#: 20297-AAV5; RRID:Addgene_20297
pAAV-hSynapsin1-FLEx-axon-GCaMP6s	Broussard et al.^120^	Cat#: 112010-AAV5; RRID:Addgene_112010
pAAV-EF1a-DIO-hChR2(H134R)-mCherry-WPRE-HGHpA	It was a gift from Dr. Karl Deisseroth	Cat #: 20297-AAV5; RRID:Addgene_20297
Critical commercial assays		
Choline/Acetylcholine Assay kit	Abcam	Cat#: ab65345
Experimental models: Organisms/strains		
eIF4E^*wt/βtEif4ε*^	Ruggero et al.^121^ Santini et al.^44^	Kindly provided by Dr. Eric Klann
VGLUT3-*Cre*	IMSR, JAX	RRID:IMSR_JAX:018147
DAT-*Cre*	Ekstrand et al.^75^ Stagkourakis et al.^76^	Kindly provided by Dr. Gilberto Fisone
Oligonucleotides		
Primers for eIF4E Tg amplification: 5′-CACAGCTACAAAGAGCGGCTCCACC -3′ 5′-CACTGCATTCTAGTTGTGGTTTGTCC-3′	Thermo Fisher Scientific	N/A
Primers for VGLUT3-*Cre* amplification: 5′-ACACCGGCCTTATTCCAAG-3′ 5′-AGATGTCTTATGGAGCCACCAC-3′ 5′-CTGAGACCAAGGTCCATATTCC-3′	Thermo Fisher Scientific	N/A
Primers for DAT-*Cre* amplification: 5′-CATGGAATTTCAGGTGCTTGG-3′ 5′-ATGAGGGTGGAGTTGGTCAG-3′ 5′-CGCGAACATCTTCAGGTTCT-3′	Thermo Fisher Scientific	N/A
Software and algorithms		
GraphPad Prism (v10)	GraphPad Software	https://www.graphpad.com RRID:SCR_002798
ImageJ	NIH	https://imagej.net RRID:SCR_003070
pCIamp 11	Molecular Devices	http://www.moleculardevices.com/products/software/pclamp.html RRID:SCR_011323
Stimfit0.15	Guzman et al.^122^	https://github.com/neurodroid/stimfit RRID:SCR_016050
Image Studio Lite	Li-COR	http://www.licor.com/bio/products/software/image_studio_lite/RRID:SCR_013715
Other		
KAPA2G Fast Ready Mix	Merck	Cat#: KK5101

## Highlights

- eIF4E Tg mice exhibit impaired reversal learning
- eIF4E Tg mice display reduced evoked dopamine release in the striatum
- Dopamine deficits are triggered by acetylcholine nicotinic receptor dysfunction
- Dysregulated striatal dopamine release may drive ASD-like behavioral inflexibility
