# Supplementary material for: Dysregulated acetylcholine-mediated dopamine neurotransmission in the eIF4E Tg mouse model of autism spectrum disorders
Source: Cell Rep. Author manuscript; Available in PMC 2026 Jan 7. (PMC12775367; doi:10.1016/j.celrep.2024.114997)
Supplement: NIHMS2124379 Supplemental Material [file NIHMS2124379-supplement-NIHMS2124379_Supplemental_Material.pdf]

Cell Reports, Volume *43*

Supplemental information

Dysregulated acetylcholine-mediated dopamine neurotransmission in the eIF4E Tg mouse model of autism spectrum disorders

Josep Carbonell-Roig, Alina Aaltonen, Karin Wilson, Maya Molinari, Veronica Cartocci, Avery McGuirt, Eugene Mosharov, Jan Kehr, Ori J. Lieberman, David Sulzer, Anders Borgkvist, and Emanuela Santini

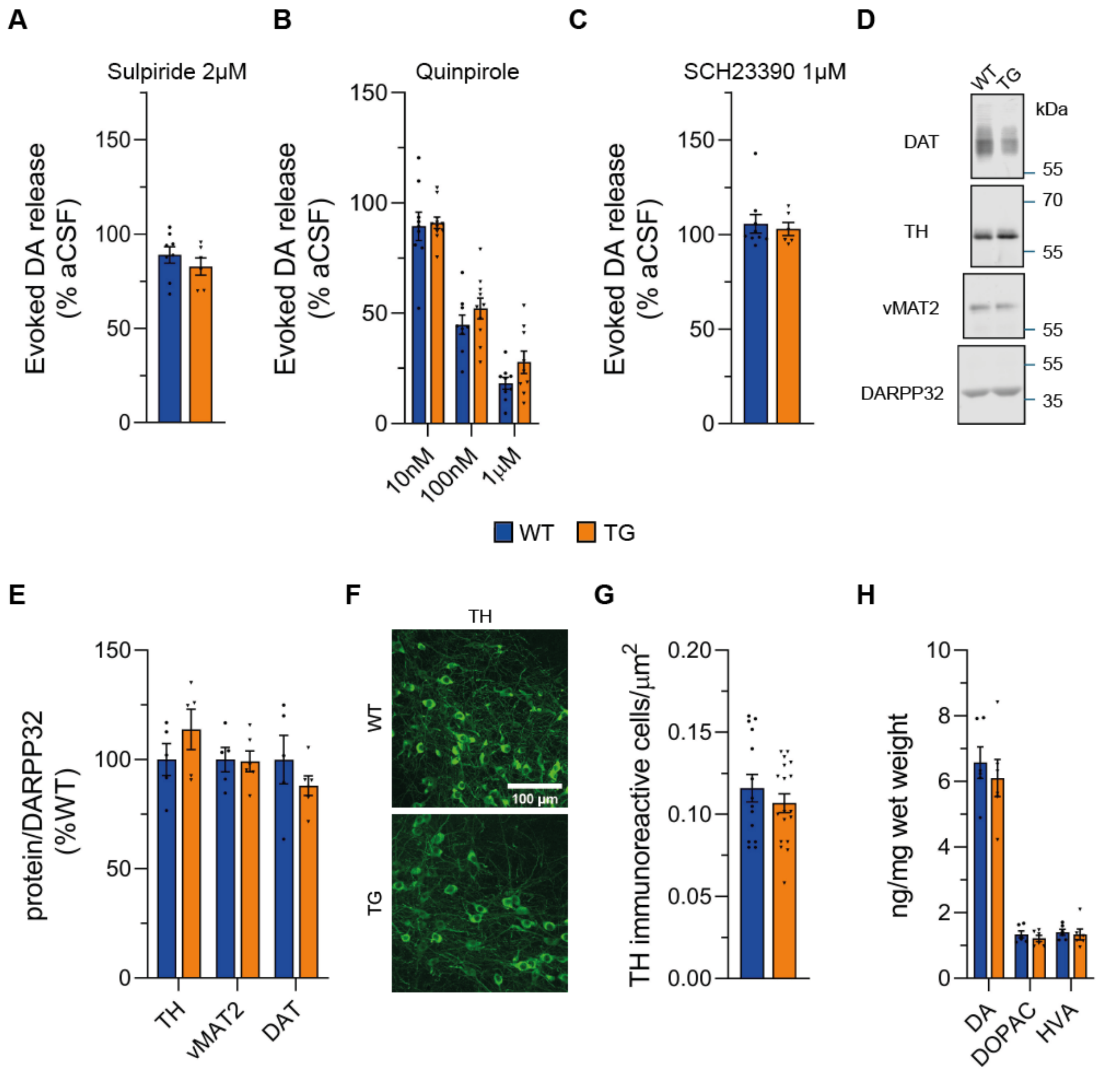

Figure S1. Intact presynaptic dopamine D2 and D1 receptor function, striatal DA biochemistry and density of DA cell bodies in the eIF4E Tg mice. (A) Peak concentrations of evoked DA release in the presence of the dopamine D2 receptors (D2R)-like antagonist sulpiride (2 μM) expressed as percentage of the baseline in aCSF. Unpaired, two-tailed t-test, t_{14}=1,808, ns=not significant. *n*= 8 slices/ 3 mice/ genotype. **(B)** Peak concentrations of evoked DA release in the presence of the D2R-like agonist quinpirole (10 nM, 100 nM and 1 μM) expressed as percentage of the baseline in aCSF. Two-way RM ANOVA, Genotype: $F_{(1,18)}$=1.26, ns; Treatment: $F_{(1.796,\ 29.63)}$=244.5, ****p<0.0001; Genotype:Treatment: $F_{(2,\ 33)}$=0.7591, ns. *n*= 8-11 slices/ 3 mice/ genotype. **(C)** Peak concentrations of evoked DA release in the presence of the dopamine D1 receptors (D1R)-like antagonist SCH23390 (1 μM) expressed as percentage of the baseline in aCSF. Unpaired, two-tailed t-test, t_{13}=0.4065, ns. *n*= 6-9 slices/ 3 mice/ genotype. **(D)** Representative Western Blot (WB) images and **(E)** quantification of tyrosine hydroxylase (TH), vesicular monoamine transporter2 (vMAT2) and dopamine reuptake transporter (DAT) in striatal homogenates. Proteins levels were expressed as ratio of DARPP-32 and as a percentage of wild-type controls (WT). TH: Unpaired, two-tailed t-test, t_9=1.6, ns.; vMAT2: Unpaired, two-tailed t-test, t_9=0.1, n.s.; DAT: Unpaired, two-tailed t-test, t_9=1.1, n.s. *n*=5-6 mice/genotype. **(F)** Representative confocal images (scale bar: 100μm) and **(G)** quantification of the number of midbrain neurons immunolabelled for TH per μm^2. Unpaired, two-tailed t-test, t_{30}=0.9, ns. *n*= 14- 18 slices/ 4 mice/ genotype. **(H)** Quantification of DA and its metabolites DOPAC and HVA in striatal tissues obtained with liquid chromatography tandem mass spectrometry (UHPLC-MS/MS). DA: Unpaired, two-tailed t-test, t_{10}=0.6, ns.; DOPAC: Unpaired, two-tailed t-test, t_{10}=0.9; n.s. HVA: Unpaired, two-tailed t-

test, t_{10}=0.3, n.s.; *n*= 6 mice/ genotype. For all graphs, bars represent group averages expressed as mean ± SEM and dots represent values for individual slices (A-C and F-G) or mice (D-E and H). Descriptive statistics for non-significant graphs are provided in Supplemental Table 6.

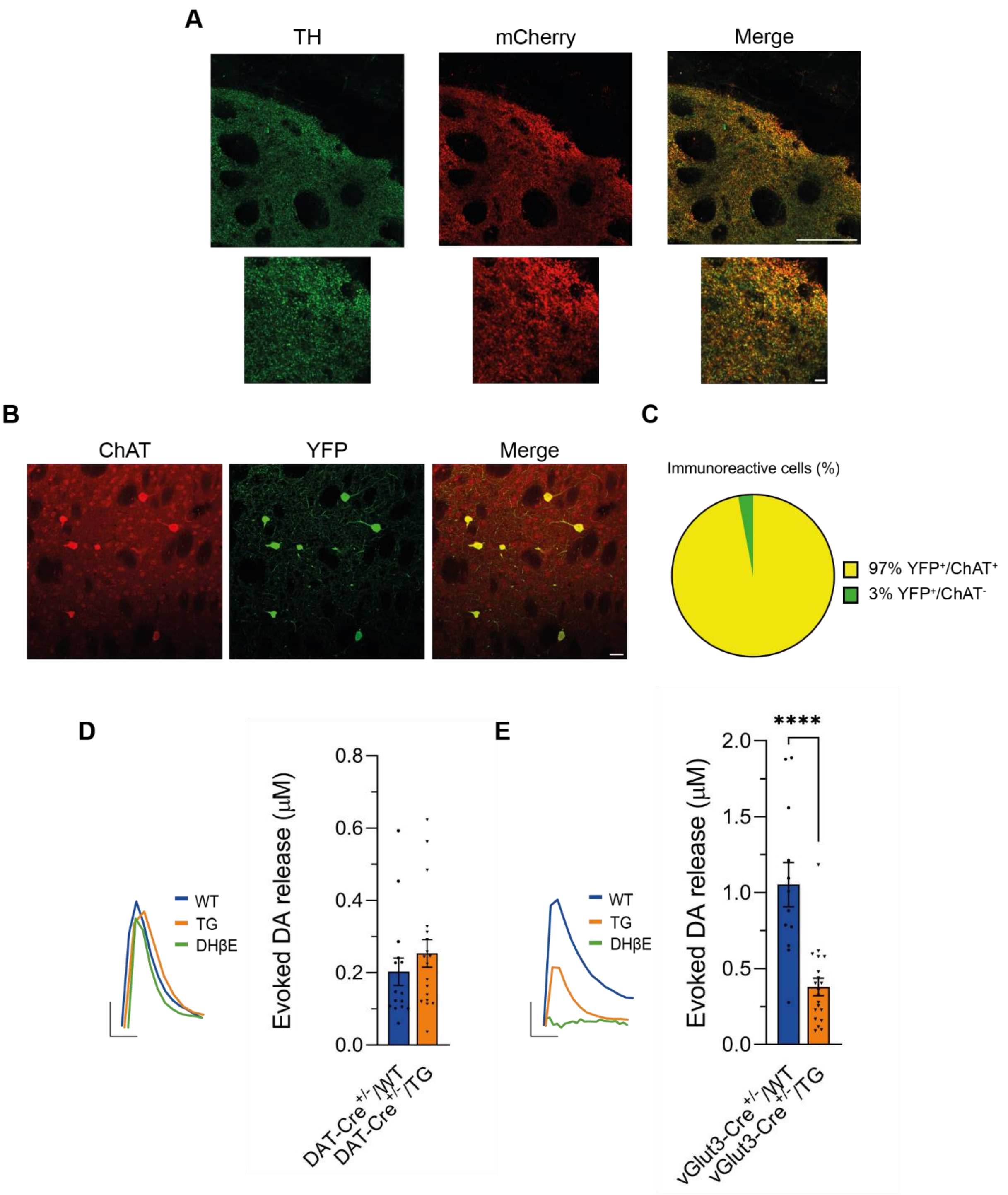

Figure S2. Functional Cre expression is confined in striatal DA axons and ACh interneurons in DAT-Cre and VGLUT3-Cre mice, respectively. (**A**) Top row: representative confocal images of the striatum of a DAT-Cre mouse injected with AAV5-EF1a-DIO-hChR2(H134R)-mCherry-WPRE-HGHpA and stained with antibodies against TH and Red Fluorescent Protein (RFP), scale bar: 100 µm. Bottom row: high magnification inserts from top row, DA terminals stained by TH colocalize with RFP labelling mCherry expressed by the AAV5, scale bar: 20 µm. (**B**) Representative confocal images of the striatum of a VGLUT3-Cre mouse injected with AAV5-Ef1a-DIO-EYFP and stained with antibodies against Green Fluorescent Protein (GFP) and choline acetyltransferase (ChAT) expressed by ACh interneurons, scale bar: 30 µm. (**C**) Representative pie chart illustrating the percentage of Yellow Fluorescent Protein positive (YFP+) neurons also positive (ChAT+) or negative (ChAT-) for ChAT. (**D**-**E**) Representative traces of FSCV recordings (left) summarized in the corresponding graphs (right), showing peak concentrations of evoked DA release

following a 5 pulse at 40Hz (5p40Hz) train stimulation of channelrhodopsin 2 (ChR2) expressed in DA terminals (**D**) or ACh interneuron (**E**). Traces are shown for wild-type (WT, blue), eIF4E Tg (TG, orange), DHβE (1 μM, green). Scale bar: 500 ms (x) and 0.1 μM (y) (**D**) and 500 ms (x) and 0.2 μM (y) (**E**). DHβE traces are included to demonstrate the dependency of DA release on ACh-nAChR in (**E**) but not (**D**). Unpaired, two-tailed *t*-test for (**D**) t_{31}=0.9336, ns=not significant. *n*= 15-18 slices/ 4 mice/ genotype; and (**E**) t_{30}=5.031, ****p<0.0001. *n*=12-20 slices/4mice/genotype. For all graphs, bars represent group averages expressed as mean ± SEM and dots represent values for individual slices. Descriptive statistics for non-significant graphs are provided in Supplemental Table 7.

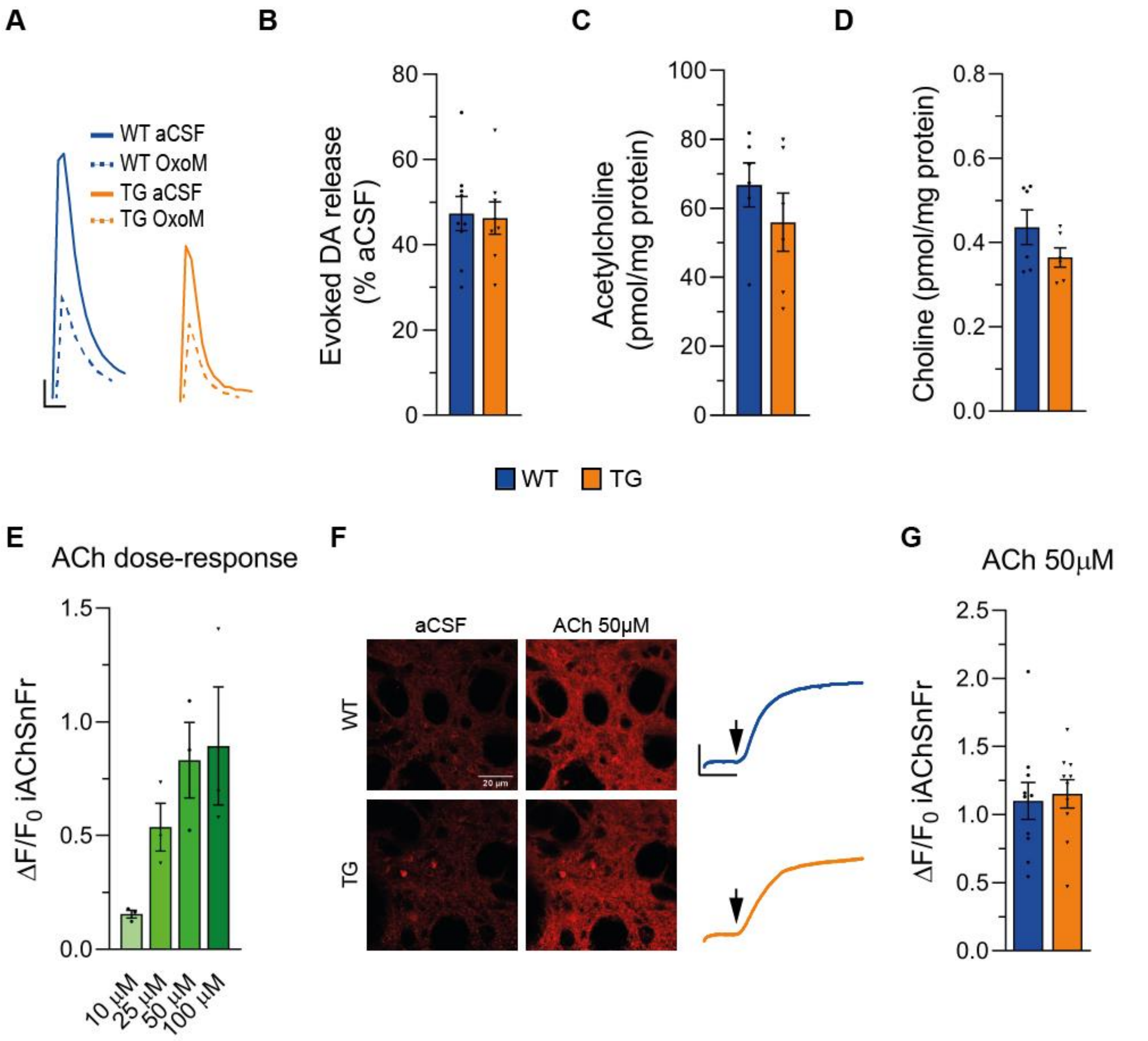

Figure S3 Preserved presynaptic muscarinic receptor function, striatal ACh levels and iAChSnFr sensitivity in the eIF4E Tg mice. (**A**-**B**) FSCV recordings in the presence of oxotremorine M (OxoM 10 µM), an agonist of muscarinic receptors, in the dorsal striatum of acute corticostriatal slices. (**A**) Representative traces of FSCV recordings summarized in (**B**); wild-type (WT, blue) and eIF4E Tg (TG, orange) before (aCSF, continuous lines) and after bath application of OxoM (OxoM, dotted lines). Scale bar: 500 ms (x) and 0.1 µM (y). (**B**) Peak concentrations of striatal evoked DA release in the presence of OxoM (10 µM) expressed as percentage of the baseline in aCSF. Unpaired, two-tailed *t*-test, t_{15}=0.19, ns=not significant. *n*= 10-12 slices/ 3 mice/ genotype. (**C**) Quantification of striatal acetylcholine and (**D**) choline in striatal tissues. Unpaired, two-tailed *t*-test, t_{43}=0.6, ns. *n*=6 mice/genotype. (**E**) Quantification of the changes in fluorescence of the iAChSnFr in striatal slices of wild-type mice following bath application of ACh at increasing concentrations (10, 25, 50, 100 µM). One-way RM ANOVA $F_{(1.6,\ 3.2)}$ = 8.2, p=0.05. *n*= 3 slices/2 mice. (**F**) Representative images (left) and traces (right) of iAChSnFr fluorescence in striatal slices of wild-type (WT, blue) and eIF4E Tg mice (TG, orange) following bath application of ACh (50 µM), indicated by black arrows. Scale bars: images 20 µm, traces 60s (x) and 30% $\Delta F/F_0$ (y). (**G**) Quantification of the changes in fluorescence of the biosensor iAChSnFr in striatal slices of WT and TG mice following application of ACh (50 µM). Unpaired, two-tailed *t*-test, t_{18}=0.3, ns. *n*= 10 slices /2mice /genotype. For all graphs, bars represent group averages expressed as mean ± SEM and dots represent values for individual slices (A-B and E-G) or mice (C-D). Descriptive statistics for non-significant graphs are provided in Supplemental Table 8.

Supplemental Table S1: related to Figure 1

Discrimination phase							
	WT			TG			
	Mean	SEM	*n* (mice)	Mean	SEM	*n* (mice)	p-value/ test
Trials to criterion	14.71	1.304	7	14.29	0.968	7	0.796 Unpaired t-test
Total errors	6.268	1.063	7	5.857	0.857	7	0.759 Unpaired t-test
Rewards	8.286	0.360	7	8.429	0.429	7	0,802 Unpaired t-test
Reversal phase							
	WT			TG			
	Mean	SEM	*n* (mice)	Mean	SEM	*n* (mice)	p-value/ test
Trials to criterion after first correct choice	8.571	0.6117	7	10.00	1.574	7	0.414 Unpaired t-test
Rewards	8.857	0.261	7	8.571	0.297	7	0.483 Unpaired t-test

Supplemental Table S2: Related to Figure 2

Dopamine release properties									
	WT				TG				
	Mean	SEM	*n* (cells/ slices)	*n* (mice)	Mean	SEM	*n* (cells/ slices)	*n* (mice)	p-value/ test
Single pulse (Evoked DA, μM)	0.763	0.063	16	5	0.469	0.034	16	6	Genotype: $F_{(1, 30)}$ = 15.99, p-val=**0.0004** ;Stimulus: $F_{(1,666, 48,30)}$ = 32.18, p-val **< 0.0001** ;Genotype x Stimulus: $F_{(2, 58)}$ = 2.579, p-val= 0.0846. Two-way RM ANOVA
5 pulses 10Hz (Evoked DA, μM)	0.916	0.081	16	5	0.561	0.044	16	6	
5 pulses 100Hz (Evoked DA, μM)	0.949	0.107	14	5	0.586	0.033	16	6	
Tau (s)	0.329	0.019	17	3	0.323	0.026	17	4	0.8556 Unpaired t-test
Ratio 10p10Hz/ Single Pulse	1.192	0.023	16	5	1.207	0.043	16	6	Genotype: $F_{(1, 30)}$ = 0.04331, p-val= 0.8366; Stimulus: $F_{(1, 28)}$ = 6.718, p-val= **0.0150**; Genotype x Stimulus: $F_{(1, 28)}$ = 0.03162, p-val= 0.8601 2way-RM-ANOVA
Ratio 10p100Hz/ Single Pulse	1.272	0.040	14	5	1.280	0.045	16	6	
Electrophysiological properties of dopamine neurons									
	WT				TG				
	Mean	SEM	*n* (cells/ slices)	*n* (mice)	Mean	SEM	*n* (cells/ slices)	*n* (mice)	p-value/ test
Firing frequency (Hz)	3.701	0.392	15	3	4.482	0.406	17	3	0.179 Unpaired t-test
Variation coeffiecient	0.2485	0.077	15	3	0.1773	0.032	17	3	0.3743 Unpaired t-test
Capacitance (pF)	118.5	9.48	19	3	115.5	6.55	27	3	0.787 Unpaired t-test
Input resistance (mOhms)	278.1	24.46	19	3	270.3	16.71	27	3	0.784 Unpaired t-test
Rheobase (pA)	77.631	21.570	19	3	77.08	25.836	25	3	0.987 Unpaired t-test
Action potential duration (ms)	1.628	0.062	19	3	1.849	0.090	26	3	0.069 Unpaired t-test
Input-Frequency curve									
	WT				TG				
Current (pA)	Mean	SEM	*n* (cells/ slices)	*n* (mice)	Mean	SEM	*n* (cells/ slices)	*n* (mice)	p-value/ test

-200	0	0	19	3	0	0	27	3	Genotype: $F_{(1,53)}$=3.434, p-val=0.069;<br><br>Current step: $F_{(3.228,171.1)}$= 93.24, p-val **<0.0001**;<br><br>Genotype x Current step: $F_{(19,1007)}$=1.808, p-val=**0.0180** /<br><br>2way-RM-ANOVA
-150	0	0	19	3	0	0	27	3	
-100	0.105	0.105	19	3	0.148	0.103	27	3	
-50	0.947	0.415	19	3	0.963	0.375	27	3	
0	2.211	0.549	19	3	2.741	0.514	27	3	
50	4.737	0.438	19	3	4.963	0.39	27	3	
100	5.789	0.647	19	3	6.667	0.489	27	3	
150	6.211	0.748	19	3	7.481	0.628	27	3	
200	6.421	0.846	19	3	8.37	0.827	27	3	
250	6.316	0.827	19	3	7.926	0.934	27	3	
300	6.105	0.924	19	3	7.111	1.026	27	3	
350	6.105	0.936	19	3	6.963	1.032	27	3	
400	5.789	0.877	19	3	6.519	1.005	27	3	
450	5.684	0.813	19	3	6.37	1.007	27	3	
500	5.263	0.783	19	3	6.296	0.898	27	3	
550	5.158	0.618	19	3	5.778	0.895	27	3	
600	5.474	0.821	19	3	5.037	0.809	27	3	
650	5.053	0.655	19	3	4.741	0.693	27	3	

Supplemental Table S3: Related to Figure 3

Photostimulation of DA axons: Input-Output Curve									
	DAT-Cre/WT				DAT-Cre/TG				
Light intensity (mW)	Mean	SEM	*n* (cells/ slices)	*n* (mice)	Mean	SEM	*n* (cells/ slices)	*n* (mice)	p-value/ test
0.2	0.028	0.005	12	4	0.024	0.004	14	4	Genotype: $F_{(1, 24)}$= 0.5952, p-val=0.4479; Stimulus: $F_{(1.310, 31.44)}$= 162.4, **p-val<0.0001**; Genotype x Stimulus: $F_{(4, 96)}$= 0.6112, p-val=0.6555 / 2way-RM-ANOVA
0.3	0.083	0.010	12	4	0.107	0.011	14	4	
0.5	0.185	0.021	12	4	0.213	0.023	14	4	
0.9	0.247	0.025	12	4	0.279	0.027	14	4	
2.0	0.293	0.031	12	4	0.310	0.026	14	4	

Supplemental Table S4: Related to Figure 4

Electrophysiological properties of cholinergic interneurons									
	VGLUT3-Cre$^{+/-}$/WT				VGLUT3-Cre$^{+/-}$/TG				
	Mean	SEM	*n* (cells/ slices)	*n* (mice)	Mean	SEM	*n* (cells/ slices)	*n* (mice)	p-value/ test
Firing frequency (Hz)	5.434	0.514	59	8	4.593	0.862	28	9	0.1787 Unpaired t-test
Variation coefficient	0.356	0.031	59	8	0.392	0.048	28	9	0.3743 Unpaired t-test
Capacitance (pF)	112.4	9.006	32	4	112.3	6.925	32	4	0.989 Unpaired t-test
Input resistance (mOhms)	143.4	10.07	32	4	167.1	8.799	32	4	0.0821 Unpaired t-test
Rheobase (pA)	-38.527	7.404	33	4	-41.209	4.998	32	4	0.7664 Unpaired t-test
Action potential duration (ms)	1.095	0.0298	33	4	1.175	0.031	32	4	0.0693 Unpaired t-test
Input-Frequency curve									
	VGLUT3-Cre$^{+/-}$/WT				VGLUT3-Cre$^{+/-}$/TG				
Current (pA)	Mean	SEM	*n* (cells/ slices)	*n* (mice)	Mean	SEM	*n* (cells/ slices)	*n* (mice)	p-value/ test
-200	0.063	0.063	32	4	0.000	0.000	33	4	Genotype: $F_{(1, 63)}$=3.820, p-val= 0.0551; Current step: $F_{(2.123, 133.7)}$= 97.00, p-val **<0.0001;** Genotype x Current step: $F_{(17, 1071)}$= 0.5234, p-val= 0.9427 / 2way-RM-ANOVA
-150	0.500	0.284	32	4	0.000	0.000	33	4	
-100	1.188	0.553	32	4	0.121	0.121	33	4	
-50	3.688	0.876	32	4	0.909	0.315	33	4	
0	10.000	0.950	32	4	6.545	0.681	33	4	
50	16.938	1.125	32	4	13.939	0.927	33	4	
100	22.250	1.442	32	4	19.273	1.282	33	4	
150	27.313	1.531	32	4	23.152	1.679	33	4	
200	29.938	1.826	32	4	26.061	1.880	33	4	
250	30.750	2.208	32	4	26.303	1.929	33	4	
300	31.563	2.485	32	4	26.485	2.136	33	4	
350	31.938	2.819	32	4	26.485	2.316	33	4	
400	30.375	2.906	32	4	27.030	2.425	33	4	
450	29.563	3.000	32	4	26.303	2.650	33	4	
500	28.688	3.112	32	4	25.758	2.570	33	4	
550	28.250	3.182	32	4	25.212	2.549	33	4	
600	27.188	3.216	32	4	22.606	2.206	33	4	
650	27.500	3.357	32	4	20.667	1.984	33	4	
Acetylcholine release (iAChSnFr)									
	WT				TG				
	Mean	SEM	*n* (cells/ slices)	*n* (mice)	Mean	SEM	*n* (cells/ slices)	*n* (mice)	p-value/test

Single pulse ($\Delta F/F_0$ %)	13.753	2.760	13	4	17.458	1.871	16	4	Genotype: $F_{(1,27)}$ = 0.5902 ,p-val=0.449; Stimulus: $F_{(1.036, 27.97)}$ = 36.68, p-val**<0,0001** ; Genotype x Stimulus: $F_{(2,54)}$ = 0.02027,p-val=0.979/ 2way-RM-ANOVA
10p10Hz ($\Delta F/F_0$ %)	21.736	3.602	13	4	24.661	2.448	16	4	
10p40Hz ($\Delta F/F_0$ %)	30.633	5.634	13	4	34.045	3.845	16	4	

Supplemental Table S5: Related to Figure 5

Electrical stimulation									
	WT				TG				
	Mean	SEM	*n* (cells/ slices)	*n* (mice)	Mean	SEM	*n* (cells/ slices)	*n* (mice)	p-value/ test
Single pulse (Evoked DA, µM)	0.958	0.089	31	6	1.163	0.157	32	7	Genotype: $F_{(1, 61)}$= 0.7065, p-val= 0.4039; Stimulus: $F_{(1, 61)}$= 152.9, p-val **<0.0001**; Genotype x Stimulus: $F_{(1, 61)}$= 0.5063, p-val=0.4795/ 2way-RM-ANOVA
5 pulses, 40Hz (Evoked DA, µM)	1.546	0.135	31	6	1.687	0.192	32	7	
**Optical stimulation**									
	vGlut3-Cre/WT				vGlut3-Cre/TG				
	Mean	SEM	*n* (cells/ slices)	*n* (mice)	Mean	SEM	*n* (cells/ slices)	*n* (mice)	p-value/ test
Single Pulse (Evoked DA, µM)	0.67	0.062	18	3	0.645	0.056	17	3	Genotype: $F_{(1, 35)}$= 0.1698, p-val=0.6828; Stimulus: $F_{(1,24)}$=6.807, p-val=**0.0154**; Genotype x Stimulus: $F_{(1,24)}$=7.642e-005 , p-val=0.9931/ 2way-RM-ANOVA
5pulses, 40Hz (Evoked DA, µM)	0.536	0.048	13	3	0.506	0.049	11	3	

Supplemental Table S6: Related to Supplemental Figure S1

Pharmacology FSCV									
	WT				TG				
	Mean	SEM	*n* (cells/ slices)	*n* (mice)	Mean	SEM	*n* (cells/ slices)	*n* (mice)	p-value/ test
Sulpiride 2µM (Evoked DA, % aCSF)	88.86	4.35	8	3	76.52	5.256	8	3	0.0921 Unpaired t-test
Quinpirole 10nM (Evoked DA, % aCSF)	89.485	6.411	9	3	91.069	2.615	11	3	Genotype: $F_{(1,18)}$=1.26, p-val=0.2764; Dose: $F_{(1.796,29.63)}$= 244.5, p-val **<0.0001**; Genotype x Dose: $F_{(2,33)}$=0.7591, p-val=0.4761/ 2way-RM-ANOVA
Quinpirole 100nM (Evoked DA, % aCSF)	44.862	4.305	9	3	52.159	4.73	10	3	
Quinpirole 1µM (Evoked DA, % aCSF)	18.281	2.669	9	3	27.856	5.068	9	3	
SCH 23390 1µM (Evoked DA, % aCSF)	105.7	4.978	9	3	103	3.401	6	3	0.6910 Unpaired t-test
Western-Blot									
	Mean	SEM	*n* (cells/ slices)	*n* (mice)	Mean	SEM	*n* (cells/ slices)	*n* (mice)	p-value/ test
TH (% of WT)	100	7.311	-	5	113.806	9.245	-	5	0.1530 Unpaired t-test
vMAT2 (% of WT)	100	5.583	-	5	99.192	4.722	-	5	0.9138 Unpaired t-test
DAT (% of WT)	100	11.101	-	5	88.079	4.534	-	6	0.3150 Unpaired t-test
Immunohistochemistry									
	Mean	SEM	*n* (cells/ slices)	*n* (mice)	Mean	SEM	*n* (cells/ slices)	*n* (mice)	p-value/ test
Tyrosine Hydroxylase positive cells density (cells/µm^2)	0.1161	0.0084	14	4	0.1069	0.0057	18	4	0.3557 Unpaired t-test
Ultra-high performance liquid chromatography-tandem mass spectrometry (UHPLC-MS/MS)									
	Mean	SEM	*n* (cells/ slices)	*n* (mice)	Mean	SEM	*n* (cells/ slices)	*n* (mice)	p-value/ test
Dopamine (ng/ mg tissue)	6.576	0.479	-	6	6.105	0.568	-	6	0.5407 Unpaired t-test

DOPAC (ng/ mg tissue)	1.339	0.105	-	6	1.218	0.094	-	6	0.4094 Unpaired t-test
HVA (ng/ mg tissue)	1.404	0.091	-	6	1.339	0.165	-	6	0.7372 Unpaired t-test

Supplemental Table S7: Related to Supplemental Figure S2

Photostimulation of Dopamine Terminals									
	DATCre+/-/WT				DATCre+/-/TG				
	Mean	SEM	*n* (cells/ slices)	*n* (mice)	Mean	SEM	*n* (cells/ slices)	*n* (mice)	p-value/ test
5 pulses 40Hz (Evoked DA release, µM)	0.203	0.038	15	4	0.254	0.038	18	4	0.3577 Unpaired t-test

Supplemental Table S8: Related to Supplemental Figure S3

Oxotremorine 10 µM FSCV									
	WT				TG				
	Mean	SEM	*n* (cells/ slices)	*n* (mice)	Mean	SEM	*n* (cells/ slices)	*n* (mice)	p-value/ test
(Evoked DA, % aCSF)	47.34	4.011	9	3	46.31	3.819	8	3	0.8548 Unpaired t-test
ELISA									
	WT				TG				
	Mean	SEM	*n* (cells/ slices)	*n* (mice)	Mean	SEM	*n* (cells/ slices)	*n* (mice)	p-value/ test
Acetylcholine (pmol/ mg protein)	66.81	6.411	-	6	55.99	8.451	-	6	0.332 Unpaired t-test
Choline (pmol/ mg protein)	0.436	0.041	-	6	0.3649	0.022	-	6	0.1632 Unpaired t-test
iAChSnFr sensitivity to 50 µM Acetylcholine									
	WT				TG				
	Mean	SEM	*n* (cells/ slices)	n (mice)	Mean	SEM	*n* (cells/ slices)	*n* (mice)	p-value/ test
iAChSnFR ($\Delta F/F_0$)	1.101	0.135	10	2	1.154	0.103	10	2	0.7621 Unpaired t-test
